# OGDH and Bcl-xL loss causes synthetic lethality in glioblastoma

**DOI:** 10.1172/jci.insight.172565

**Published:** 2024-03-14

**Authors:** Trang T.T. Nguyen, Consuelo Torrini, Enyuan Shang, Chang Shu, Jeong-Yeon Mun, Qiuqiang Gao, Nelson Humala, Hasan O. Akman, Guoan Zhang, Mike-Andrew Westhoff, Georg Karpel-Massler, Jeffrey N. Bruce, Peter Canoll, Markus D. Siegelin

**Affiliations:** 1 Department of Pathology and Cell Biology, Columbia University Medical Center, New York, New York, USA.; 2 Department of Biological Sciences, Bronx Community College, City University of New York, New York, USA.; 3 Department of Neurological Surgery, and; 4 Department of Neurology, Columbia University Medical Center, New York, New York, USA.; 5 Proteomics and Metabolomics Core Facility, Weill Cornell Medicine, New York, New York, USA.; 6 Department of Pediatrics and Adolescent Medicine, and; 7 Department of Neurosurgery, Ulm University Medical Center, Ulm, Germany.

**Keywords:** Oncology, Apoptosis pathways

## Abstract

Glioblastoma (GBM) remains an incurable disease, requiring more effective therapies. Through interrogation of publicly available CRISPR and RNAi library screens, we identified the α-ketoglutarate dehydrogenase (*OGDH*) gene, which encodes an enzyme that is part of the tricarboxylic acid (TCA) cycle, as essential for GBM growth. Moreover, by combining transcriptome and metabolite screening analyses, we discovered that loss of function of OGDH by the clinically validated drug compound CPI-613 was synthetically lethal with Bcl-xL inhibition (genetically and through the clinically validated BH3 mimetic, ABT263) in patient-derived xenografts as well neurosphere GBM cultures. CPI-613–mediated energy deprivation drove an integrated stress response with an upregulation of the BH3-only domain protein, Noxa, in an ATF4-dependent manner, as demonstrated by genetic loss-of-function experiments. Consistently, silencing of Noxa attenuated cell death induced by CPI-613 in model systems of GBM. In patient-derived xenograft models of GBM in mice, the combination treatment of ABT263 and CPI-613 suppressed tumor growth and extended animal survival more potently than each compound on its own. Therefore, combined inhibition of Bcl-xL along with disruption of the TCA cycle might be a treatment strategy for GBM.

## Abstract 2

Loss of alpha-ketoglutarate dehydrogenase function is synthetically lethal in combination with Bcl-xL inhibition in preclinical model systems of glioblastoma.

## Introduction

It remains unclear why isocitrate dehydrogenase–wild-type (IDH–wild-type) glioblastoma (GBM), the most common primary brain tumor in adults, remains highly resistant to therapy. There are numerous explanations for this phenomenon, including the cellular heterogeneity, the blood-brain barrier, the immunosuppressive microenvironment, and altered metabolism (1–6).

While a role for metabolism in GBM was established more than a decade ago (at the time of the discovery of IDH-mutant gliomas) (1), we are still attempting to fully appreciate the impact of tumor metabolism on GBM growth. In this context, it has been recently suggested that GBMs appear to utilize a range of different fuel sources rather than relying exclusively on glucose, which was the leading thought over the last century. In this regard, based on Otto Warburg’s theory, glucose is metabolized to lactate and in turn (to avoid glycolytic stasis), lactate is excreted into the microenvironment (7). However, we and other groups provided recent evidence that the tricarboxylic acid (TCA) cycle is pivotal for GBM growth and involves the mitochondrial production of citrate that in turn is exported to the cytosol to be converted to acetyl-CoA, which serves as the initial substrate for fatty acid and cholesterol biosynthesis (8–12). The role of the oxidative portion of the TCA cycle (starting with the IDH reaction), which involves α-ketoglutarate dehydrogenase (OGDH) among other enzymes, has remained unclear and its role in GBM growth warrants investigation.

Intrinsic apoptosis is a critical component in the response and resistance to therapy (13). At the molecular level, this process is carefully regulated by the pro- and antiapoptotic Bcl-2 family of proteins. While Bcl-xL, Bcl-2, and Mcl-1 are antiapoptotic, Noxa, BIM, BAX, and BAK are drivers of cell death. In this context, Noxa antagonizes the function of Mcl-1, and therefore high levels of Noxa render cancer cells susceptible to BH3 mimetics, such as ABT263 and ABT199, also known as navitoclax and venetoclax, respectively (14–16).

Here, through interrogation of genome-wide pooled CRISPR library screens performed in GBM models (https://depmap.org/portal/context/Glioblastoma Accessed February 1, 2023), we unexpectedly found that *OGDH* is a critical driver gene for GBM growth irrespective of their genetic background. The OGDH complex, which is composed of OGDH, dihydrolipoamide succinyltransferase (DLST), dihydrolipoamide dehydrogenase (DLD), and the assembly factor KGD4, produces succinyl-CoA and NADH from 2-oxoglutarate in an irreversible chemical reaction (oxidative decarboxylation) (17, 18). Because OGDH is critical in the context of the TCA cycle and because it was identified as a central dependency in CRISPR library screens in GBM cultures, we focused on this enzyme in our studies. Moreover, a clinically validated inhibitor of OGDH is available as well, enabling translational studies in GBM cultures and animal models. We demonstrated that loss of function of OGDH affects central carbon metabolism and activates the integrated stress response (ISR), with upregulation of proapoptotic Noxa, rendering GBM cells sensitive to Bcl-xL inhibition.

## Results

### OGDH is an oncogene that promotes GBM growth and survival.

**Figure 1:**
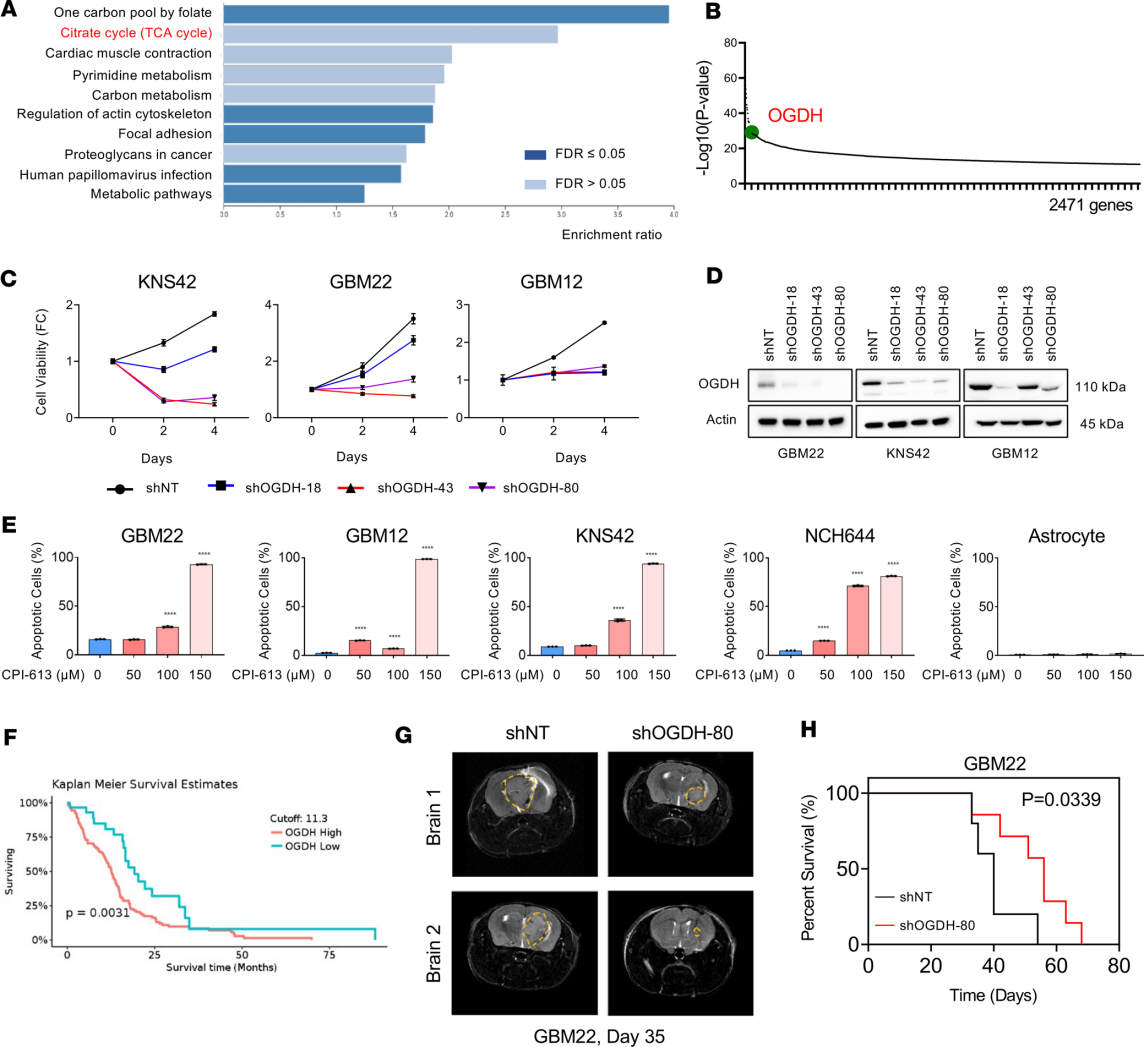
Genetic or pharmacological inhibition of OGDH, a key enzyme of the TCA cycle, reduces the growth of GBM cultures. (**A** and **B**) CRISPR and RNAi library screening (obtained and analyzed from the DepMAP database) points toward increased reliance of GBM cells on several TCA cycle enzymes, especially the *OGDH* gene. (**C**) KNS42, GBM22, and GBM12 cells were transduced with lentiviral vectors containing either nontargeting shRNA (shNT) or shRNAs against OGDH. Cellular viability analysis was performed for up to 4 days (*n* = 4 per group). FC, fold change. (**D**) Western blots of KNS42, GBM22, and GBM12 cells transduced with lentiviral vectors containing either shNT or shRNAs against OGDH. Actin was used as a loading control. (**E**) GBM22, GBM12, KNS42, NCH644, and astrocytes were treated with increasing concentrations of CPI-613 for 72 hours, labeled with Annexin V/PI dye, and analyzed by flow cytometry for apoptosis induction (*n* = 3 per group). (**F**) Shown is the survival curve of patients (wild-type and mutated IDH) with high or low mRNA levels of OGDH from TCGA database. Cutoff point (maximally selected rank statistics) was calculated through GlioVis (http://gliovis.bioinfo.cnio.es/ Last accessed March 26, 2024.), which yielded a cutpoint of 11.3 for mRNA. High levels of OGDH correlate with a worse overall survival in patients. (**G** and **H**) GBM22 cells were transduced with lentiviral vectors containing either shNT or shRNAs against OGDH, and were implanted in the right striatum of nude mice. (**G**) Representative MRI images of brain tumors from the experiment in **H** are shown (Bruker BioSpec, 9.4 Tesla). (**H**) The log-rank test was used to assess statistical significance (*n* = 5 in shNT and *n* = 7 in shODGH-80). Median survival was 40 days in GBM22 with shNT and 56 days in GBM22 with shOGDH-80. Statistical significance was assessed by 1-way ANOVA with Dunnett’s multiple-comparison test (**E**) or 2-tailed Student’s *t* test (**H**). Data are shown as mean ± SD. ****P < 0.001.

Analysis of publicly available CRISPR and RNAi library screens of GBM cells from the DepMAP database suggested dependency of GBM cells on the *OGDH* gene as well as on the TCA cycle (Figure 1, A and B). To confirm the involvement of OGDH in proliferation of GBM cells, we silenced the expression of this enzyme in KNS42, GBM22, and GBM12 cells through several shRNAs that specifically target *OGDH*. We observed a significant growth reduction of GBM cells with silenced OGDH (Figure 1, C and D). Following genetic interference with *OGDH*, we also found an induction of cell death in GBM22 and GBM12 cells (Supplemental Figure 1A; supplemental material available online with this article; https://doi.org/10.1172/jci.insight.172565DS1). Similar findings were observed in GBM cells treated with CPI-613, a drug that has reached phase III clinical testing and is known to interfere with the OGDH enzyme (19–21) (Figure 1E). Notably, human astrocytes revealed only a marginal response to CPI-613, suggesting a favorable toxicity profile (Figure 1E). In order to assess the prognostic implications of OGDH, we interrogated The Cancer Genome Atlas (TCGA) database (https://www.cancer.gov/ccg/research/genome-sequencing/tcga Accessed February 1, 2023) of GBM and found that high OGDH mRNA levels correlated with a worse overall survival (Figure 1F). To evaluate the effects of this gene in vivo, GBM22 cells were transduced with nontargeting or *OGDH*-specific shRNA followed by injection into nude mice. We found that mice harboring tumors derived from GBM22 cells transduced with OGDH shRNA revealed an extended overall survival as compared with the control animals (Figure 1, G and H, and Supplemental Figure 1B). Overall, this finding suggested that *OGDH* acts as an oncogene that promotes GBM growth.

### Loss of function of OGDH affects intrinsic apoptosis by increasing proapoptotic Noxa protein levels.

**Figure 2:**
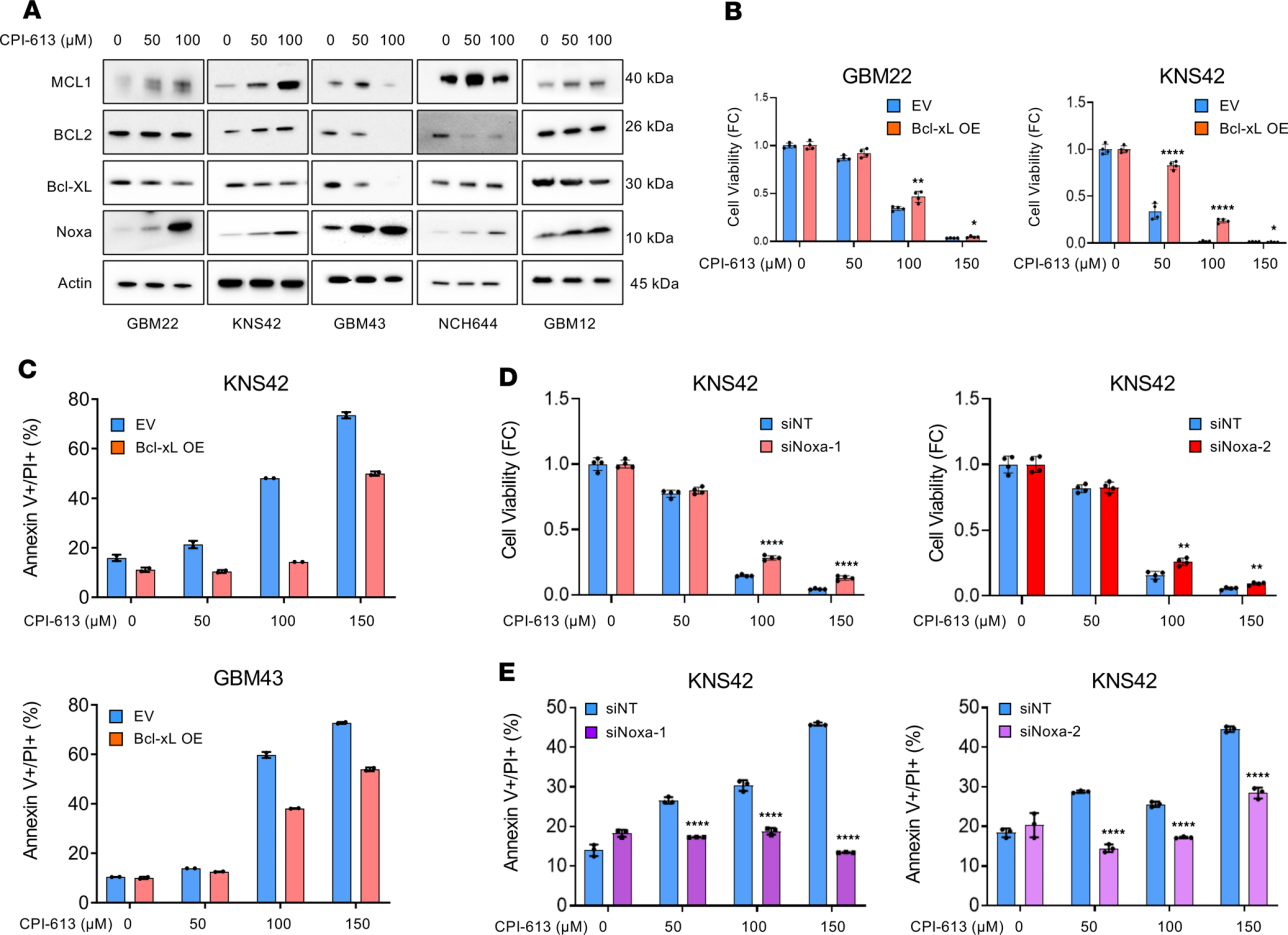
Treatment with CPI-613 increases the expression of proapoptotic Noxa and suppresses Bcl-xL levels to induce apoptosis. (**A**) GBM22, KNS42, GBM43, NCH644, and GBM12 cells were treated with increasing concentrations of CPI-613 for 24 hours and were analyzed for the Bcl-2 family members by Western blotting. (**B**) GBM22 and KNS42 cells were transduced with an empty vector (EV) or a vector containing Bcl-xL cDNA (using adenoviruses), treated with increasing concentrations of CPI-613, and cellular viability was analyzed (*n* = 4 per group). FC, fold change. (**C**) GBM43 and KNS42 cells were transduced with an EV or a vector containing Bcl-xL cDNA, treated with increasing concentrations of CPI-613, labeled with Annexin V/PI dye, and analyzed by flow cytometry (*n* = 2 per group). (**D** and **E**) KNS42 cells were transfected with nontargeting siRNA (siNT) or siRNAs against Noxa. Transfected cells were treated with CPI-613 and cellular viability analysis was performed (**D**, *n* = 4 per group), and flow cytometry following labeling with Annexin V/PI was performed (**E**, *n* = 3 per group). Statistical significance was assessed by 2-tailed Student’s *t* test (**B**, **D**, and **E**). Data are shown as mean ± SD. *P < 0.05, **P < 0.01, ****P < 0.001.

Next, we wanted to determine whether the induction of apoptosis by CPI-613 treatment is mediated by a change in expression of the Bcl-2 family of proteins. While the Mcl-1 protein levels were increased in the presence of CPI-613, we detected a reduction in Bcl-xL protein levels in different GBM cells (GBM22, KNS42, GBM43, NCH644, and GBM12) (Figure 2A). The Bcl2 protein levels seemed to be unchanged in GBM cells treated with CPI-613, except in GBM43 and NCH644 cells (Figure 2A). Notably, we observed an upregulation of the Noxa protein, a proapoptotic Bcl-2 family member, in GBM cells treated with increasing concentrations of CPI-613 (Figure 2A). In addition, we also determined the mRNA expression levels of Mcl-1, Bcl-2, and Bcl-xL in GBM22, KNS42, and GBM43 cells. Overall, we observed a reduction in Bcl-xL in response to CPI-613 treatment (Supplemental Figure 2A). In addition, we determined the expression levels of Noxa, Mcl-1, Bcl-2, and Bcl-xL following genetic loss of function of OGDH. Akin to CPI-613, we found that shRNA-mediated reduction of OGDH elicits an increase in proapoptotic Noxa in GBM22 and KNS42 cells, suggesting that Noxa might have a critical role (Supplemental Figure 2B).

To support the involvement of Bcl-xL in cell death elicited by CPI-613 treatment, we ectopically overexpressed Bcl-xL in GBM cells by using adenoviruses. First, we assessed the effect of Bcl-xL overexpression on CPI-613 treatment in the setting of a cellular viability assay. We observed a rescue of loss of viability elicited by CPI-613 treatment in Bcl-xL–overexpressing GBM cells (Figure 2B and Supplemental Figure 2C). Next, we extended our study to analyze apoptosis elicited by CPI-613 in the presence or absence of Bcl-xL overexpression in KNS42 and GBM43 cells. As anticipated, Bcl-xL overexpression mitigated cell death induced by CPI-613 (Figure 2C). These data suggest an involvement of Bcl-xL in the cell death caused by CPI-613 treatment in GBM cells.

To assess whether the upregulation of Noxa protein is required for the cell death induction mediated by CPI-613, we silenced the expression of Noxa in KNS42 and GBM12 cells (Figure 2, D and E, and Supplemental Figure 2, D–H). The knockdown of Noxa was confirmed by Western blotting (Supplemental Figure 2, E and H). We found that silencing of Noxa by 2 different siRNAs mitigated CPI-613–mediated loss of cellular viability as well as cell death in KNS42 and GBM12 (Figure 2, D and E, and Supplemental Figure 2, D–H). Notably, the effect size on cell death was more pronounced since Noxa is a primary regulator of apoptosis (14–16).

### Loss of function of OGDH or CPI-613 treatment impairs TCA cycle functionality coupled with a reduced oxygen consumption rate.

**Figure 3:**
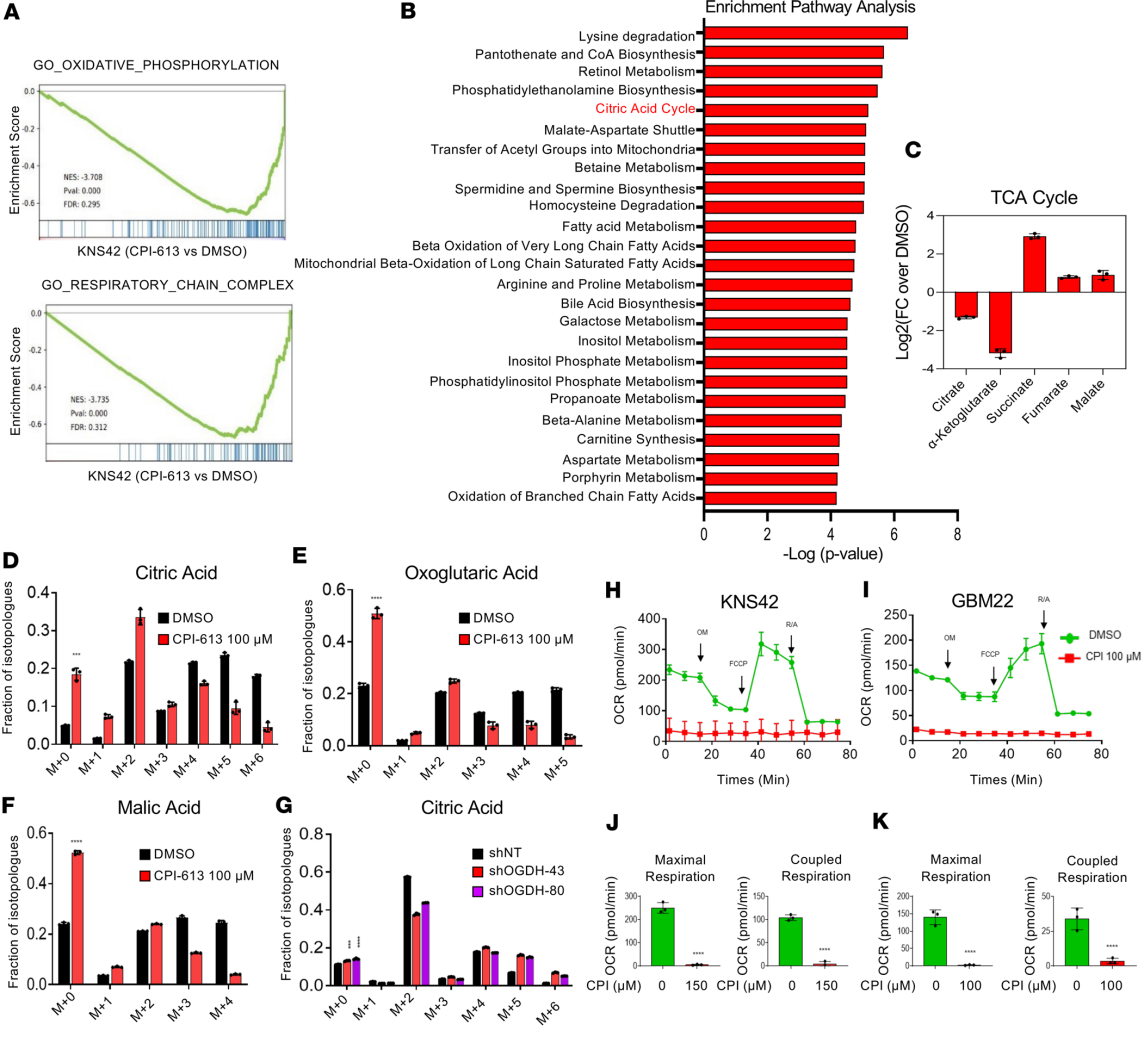
CPI-613 treatment reduces the labeling of TCA cycle metabolites from glucose and the oxygen consumption rate in GBM cells. (**A**) KNS42 cells were treated with CPI-613 for 24 hours, subjected to RNA-seq, and followed by GSEA. NES, normalized enrichment score; FDR, false discovery rate. (**B** and **C**) KNS42 cells were treated with CPI-613 for 24 hours and were processed for polar metabolite LC/MS analysis. The metabolite enrichment analysis was performed by using MetaboAnalyst (https://www.metaboanalyst.ca/). Shown is the enrichment pathway analysis and the citric acid cycle is highlighted in red. FC, fold change. (**D**–**F**) KNS42 cells were treated with 100 μM CPI-613 in DMEM containing 25 mM U-^13^C_6_-glucose, 4 mM glutamine, and 1.5% dialyzed FBS for 24 hours. Shown are fractions of the isotopologues for each metabolite (*n* = 3 per group). (**G**) GBM22 cells were transduced with either nontargeting shRNA (shNT) or shRNAs against OGDH. The transduced cells were cultured in DMEM containing 25 mM U-^13^C_6_-glucose, 4 mM glutamine, and 10% dialyzed FBS for 24 hours (*n* = 3 per group). (**H**–**K**) KNS42 and GBM22 cells were treated with CPI-613 (CPI) for 24 hours and subjected to extracellular flux analysis to analyze maximal respiration and coupled respiration in **J** and **K**. OM, oligomycin; FCCP, carbonylcyanide-4 (trifluoromethoxy) phenylhydrazone; R/A, rotenone and antimycin (*n* = 3 per group). Statistical significance was assessed by 2-tailed Student’s *t* test (**D**–**F**, **J**, and **K**) or 1-way ANOVA with Dunnett’s multiple-comparison test (**G**). Data are shown as mean ± SD. ***P < 0.001; ****P < 0.0001.

To evaluate how CPI-613 affects the transcriptome of GBM cells, we performed RNA-seq and gene set enrichment analysis (GSEA). The GSEA data revealed suppression of gene sets related to oxidative phosphorylation and respiratory chain complexes in GBM cells treated with CPI-613 (Figure 3A), in keeping with its predicted targets. We also performed ^13^C tracing analysis to study the impact of CPI-613 on the TCA cycle as well as other metabolic pathways. As anticipated, we found that CPI-613 treatment led to a substantial reduction in labeling of metabolites in the TCA cycle from glucose carbons (Figure 3, B–F, and Supplemental Figure 3, A–C). The Warburg effect includes other additional metabolic pathways such as glycolysis, the pentose phosphate pathway, and serine/amino acid synthesis. We noted a decrease in ^13^C-glucose labeling of metabolites associated with these pathways (Supplemental Figure 3, B and C). Next, we assessed how genetic loss of function of OGDH affects glucose-related labeling of citrate. We found that shRNA-mediated suppression of OGDH reduced citrate labeling from glucose and suppressed the fraction of labeling of the m+2 citrate isotopologue (Figure 3G and Supplemental Figure 3D). Other metabolites were affected as well but revealed increases in labeling, consistent with predominant “counter-clockwise” cycling via the pyruvate carboxylase reaction (Supplemental Figure 3D). Glycolytic intermediates displayed some minor changes as well (Supplemental Figure 3D).

Extracellular flux analysis also showed a suppression of the oxygen consumption rate (OCR) and ATP production mediated by loss of function of OGDH and CPI-613 treatment in KNS42 and GBM22 cells (Figure 3, H–K, and Supplemental Figure 4, A–D). To confirm that loss of energy production mediated the cell death elicited by loss of function of OGDH, we treated KNS42 and GBM22 cells with CPI-613 in the presence or absence of ATP. We detected a partial rescue from loss of cellular viability driven by CPI-613 treatment (Supplemental Figure 4E). These observations support the notion that loss of function of the OGDH enzyme blocks the TCA cycle and results in energy deprivation that in turn mediates loss of viability in GBM cells.

### Loss of function of OGDH activates the endoplasmic reticulum stress response, which facilitates Noxa upregulation in an ATF4-dependent manner.

**Figure 4:**
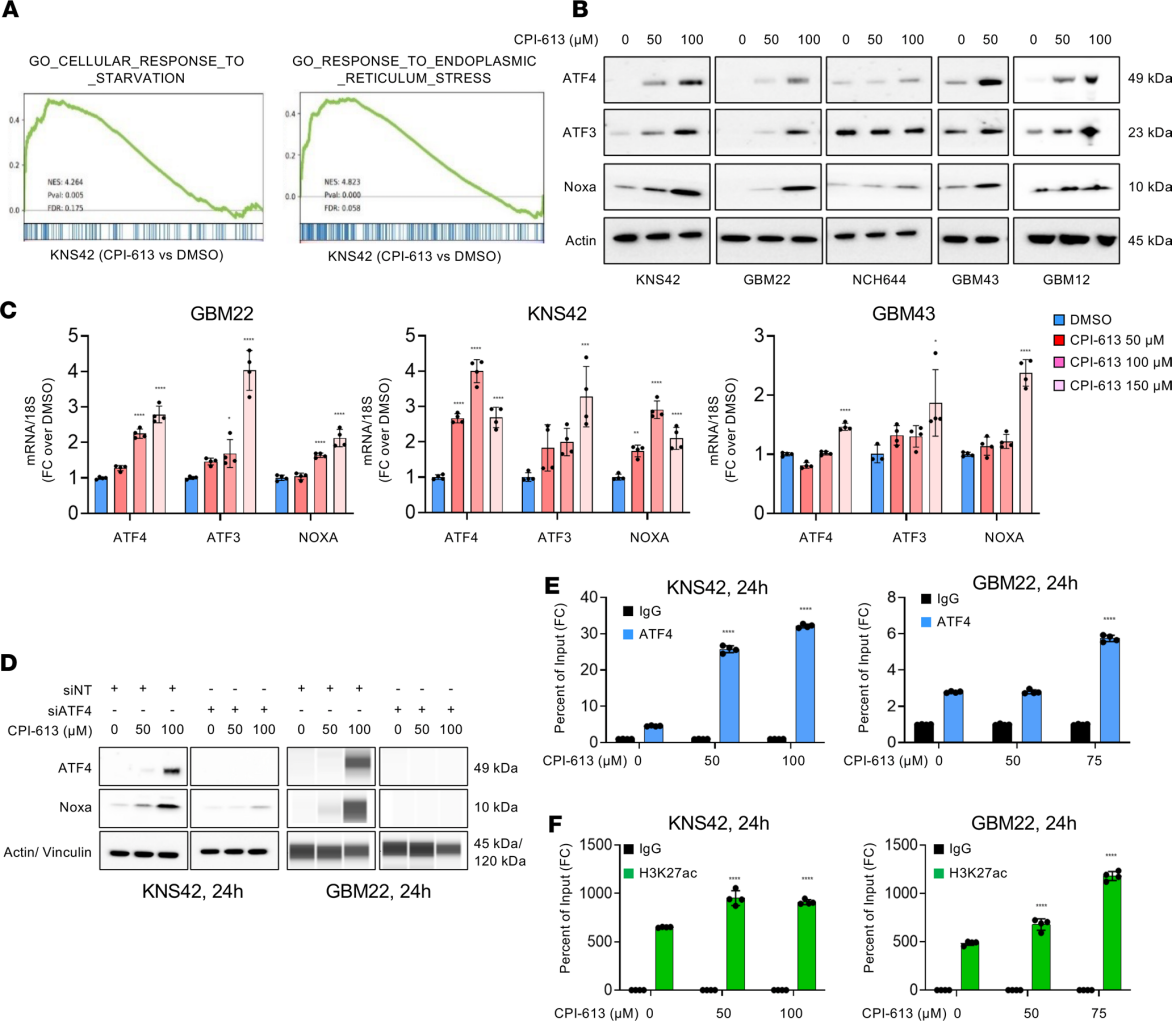
CPI-613 treatment causes energy deprivation and activates endoplasmic reticulum stress signaling. (**A**) KNS42 cells were treated with CPI-613 for 24 hours, subjected to RNA-seq, and followed by GSEA. NES, normalized enrichment score; FDR, false discovery rate. (**B**) Western blots of KNS42, GBM22, GBM43, NCH644, and GBM12 cells treated with increasing concentrations of CPI-613 for 24 hours. Actin is a loading control. (**C**) Real-time PCR analysis of GBM22, KNS42, and GBM43 cells treated with increasing concentrations of CPI-613 for 24 hours. *18S* is a housekeeping gene. FC, fold change. (**D**) Standard Western blot (KNS42) or protein capillary electrophoresis analyses (GBM22) of cells transfected with nontargeting siRNA (siNT) or with siRNA against ATF4 (total) followed by treatment with CPI-613 for 24 hours. Actin or vinculin is a loading control. (**E**) KNS42 and GBM22 cells were treated with CPI-613 for 24 hours and were subjected to chromatin immunoprecipitation with either a control antibody (IgG; negative control) or an antibody against ATF4. The Noxa region was amplified by PCR. (**F**) KNS42 and GBM22 cells were treated with CPI-613 for 24 hours and were subjected to chromatin immunoprecipitation with either a control antibody (IgG; negative control) or an antibody against H3K27ac. The Noxa region was amplified by PCR (*n* = 4 per group). Statistical significance was assessed by 1-way ANOVA with Dunnett’s multiple-comparison test (**C**) or 2-tailed Student’s *t* test (**E** and **F**). Data are shown as mean ± SD. *P < 0.05; **P < 0.01; ***P < 0.001; ****P < 0.0001.

Our transcriptome analysis and GSEA pointed toward an increase in energy deprivation and an endoplasmic reticulum stress response (Figure 4A), consistent with the findings above (Figure 3). We hypothesized that the loss of energy will lead to an activation of the ISR, with upregulation of ATF4 and ATF3 (bona fide stress response transcription factors) that in turn would affect the expression of proapoptotic Noxa. To this end, we determined the ATF3 and ATF4 protein levels following CPI-613 treatment. Our data showed an increased expression of ATF3 and ATF4 elicited by CPI-613 in KNS42, GBM12, GBM22, GBM43, and NCH644 cells (Figure 4B and Supplemental Figure 5A). We also observed an increase in ATF3 and ATF4 mRNA following treatment with CPI-613 (Figure 4C). Consistently, we detected an upregulation of Noxa mRNA and protein levels that suggests an involvement of ATF4 in regulating Noxa levels following CPI-613 treatment (Figure 4, B and C). To demonstrate that the increase in both ATF4 and Noxa is due to the loss of function of OGDH, we determined the expression levels of these proteins in GBM cells with silenced OGDH. We found that shRNA-mediated reduction of OGDH led to a consistent increase in ATF3, ATF4, and Noxa, consistent with the findings observed following CPI-613 treatment (Supplemental Figure 5B).

Next, we hypothesized that the loss of energy elicited by loss of function of OGDH led to an activation of the ISR with activation of ATF4. To address this hypothesis, we analyzed the expression of AMPK and phosphorylated AMPK (threonine 172) (Supplemental Figure 5C) and found an enhancement of AMPK phosphorylation, indicative of a decrease in ATP levels. To demonstrate that energy deprivation (mediated by loss of function of OGDH) is responsible for the upregulation of ATF4, GBM cells were treated with CPI-613 in the presence or absence of ATP. Consistently, we observed that ATP suppressed the CPI-613–mediated increase in ATF4 protein levels (Supplemental Figure 5D).

To elucidate how CPI-613 treatment led to an increase in Noxa protein and mRNA levels, we hypothesized that this is most likely related to the activation of the ISR. To test this hypothesis, we analyzed the Noxa protein levels in GBM cells that were transfected with nontargeting or ATF4-specific siRNA and treated with increasing concentrations of CPI-613. The silencing of ATF4 abrogated the increase in Noxa protein level in the presence of CPI-613 treatment in both KNS42 and GBM22 cells (Figure 4D and Supplemental Figure 5E). To confirm that binding of ATF4 to the Noxa promoter is enhanced upon treatment with CPI-613, we performed chromatin immunoprecipitation of ATF4 and H3K27ac and amplified the Noxa promoter region. Notably, we found increased binding of ATF4 on the Noxa promoter following the CPI-613 treatment (Figure 4, E and F). Consistent with the upregulation of Noxa, we detected an increased deposition of H3K27ac within the Noxa promoter as well (Figure 4F). These findings support the notion that CPI-613 activates the ISR, resulting in an increase in ATF4, which in turn binds o the Noxa promoter to upregulate Noxa mRNA and protein levels.

### Dual inhibition of Bcl-xL and OGDH elicits a synergistic reduction in viability of GBM cells.

**Figure 5:**
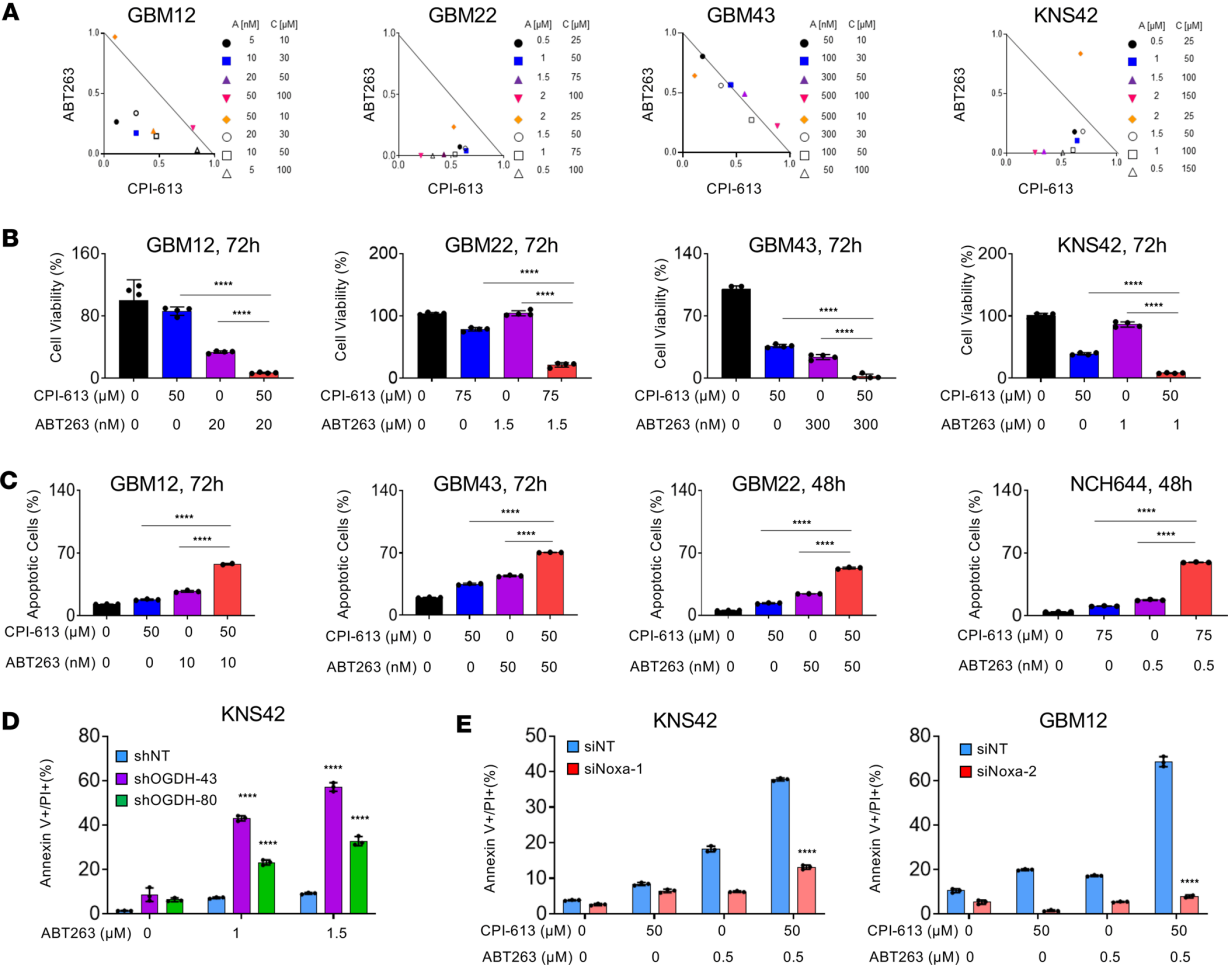
Dual inhibition with ABT263 and CPI-613 elicits a synergistic reduction in GBM cell viability. (**A** and **B**) GBM12, GBM22, GBM43, and KNS42 cells were treated with ABT263, CPI-613, or the combination of both for 72 hours, and cellular viability was analyzed. Shown are isobolograms in **A** and the quantification in **B** (*n* = 4 per group). (**C**) GBM12, GBM22, GBM43, and NCH644 cells were treated with ABT263, CPI-613, or the combination of both, labeled with Annexin V/PI dye, and analyzed by flow cytometry (*n* = 3 per group). (**D**) KNS42 cells were transduced with lentiviral vectors containing either nontargeting shRNA (shNT) or shRNAs against OGDH, treated with increasing concentrations of ABT263, and followed by flow cytometry after Annexin V/PI labeling (*n* = 3 per group). (**E**) GBM12 and KNS42 cells were transfected with nontargeting siRNA (siNT) and siRNA against Noxa. Cells were treated as indicated and labeled with Annexin V/PI followed by flow cytometry (*n* = 3 per group). Statistical significance was assessed by 1-way ANOVA with Dunnett’s multiple-comparison test (**B**–**D**) or 2-tailed Student’s *t* test (**E**). Data are shown as mean ± SD. ****P < 0.001.

Our data showed an increase in Noxa protein levels mediated by CPI-613 treatment (Figure 2A). This observation led us to hypothesize that CPI-613 and BH3 mimetics might induce a synergistic reduction in viability of GBM cells. To address this hypothesis, GBM12, GBM43, GBM22, and KNS42 cells were treated with ABT263, CPI-613, and the combination of both. ABT263 is known to inhibit both Bcl2 and Bcl-xL (15). Notably, we detected a stronger reduction in cellular viability following the drug combination treatment compared with the single treatments (Figure 5, A and B, and Supplemental Figure 6A). The GBM12 cells displayed the most synergistic growth reduction at low-nanomolar dosages for ABT263. The GBM22, GBM43, and the pediatric KNS42 cells also revealed a synergistic growth reduction following the combination treatment of CPI-613 and ABT263 (Figure 5, A and B). Next, we assessed whether the reduction in cellular viability by the combination treatment of BH3 mimetics and CPI-613 is due to the activation of apoptotic signaling pathways by performing Annexin V/PI staining. We detected a higher rate of cell death in the drug combination of ABT263 and CPI-613 in GBM12, GBM43, GBM22, and NCH644 cells (Figure 5C and Supplemental Figure 6, B and C). With regard to non-neoplastic cells (astrocytes), we found that they demonstrated a reduced susceptibility to the combination treatment as compared with the GBM cells. We also tested the combination treatment of CPI-613 with other BH3 mimetics (ABT199, a Bcl2 inhibitor and A1210477, an Mcl1 inhibitor) (22, 23). We also detected an enhanced cell death following exposure to the various drug combinations of CPI-613 and ABT199/A1210477 (Supplemental Figure 6, D–G).

To demonstrate that indeed the loss of function of OGDH is critical for the susceptibility of GBM cells to BH3 mimetics, we specifically silenced OGDH. Silencing of OGDH by siRNA or shRNA led to an enhanced cell death in the presence of ABT263 treatment as compared with nontargeting siRNA or shRNA (Figure 5D and Supplemental Figure 6, H–K). Similarly, silencing of Bcl-xL or siMcl1 mediated an enhanced cell death in the presence of CPI-613 treatment (Supplemental Figure 7, A–F). All in all, these observations suggest a synergistic growth reduction by the combination treatment and that these effects are specific to the loss of function of the relevant targets (i.e., OGDH and Bcl-xL).

We wondered whether the cell death elicited by ABT263 and CPI-613 is mediated by the activation of caspases. To investigate this, we performed Annexin V/PI analysis of the drug combination treatment in the presence or absence of the pan-caspase inhibitor, Z-VAD-FMK. We found that Z-VAD-FMK partially rescued from the cell death mediated by the combination treatment of CPI-613 and ABT263 in GBM12 and GBM22 cells (Supplemental Figure 8, A and B). Western blot analysis of GBM cells also showed an enhanced cleavage of initiator caspase-9, effector capase-3, and cleavage of PARP upon exposure to the combination treatment (Supplemental Figure 8C).

Next, we determined whether Noxa upregulation plays a pivotal role in the cell death induction by the drug combination treatment of ABT263 and CPI-613 by performing Annexin V/PI staining. Our data indicated that silencing of Noxa substantially mitigated the effect of drug combination treatment to induce cell death in KNS42 and GBM12 (Figure 5E and Supplemental Figure 8D).

### Dual inhibition of Bcl-xL and OGDH extends animal survival in orthotopic patient-derived xenograft models of human GBM.

**Figure 6:**
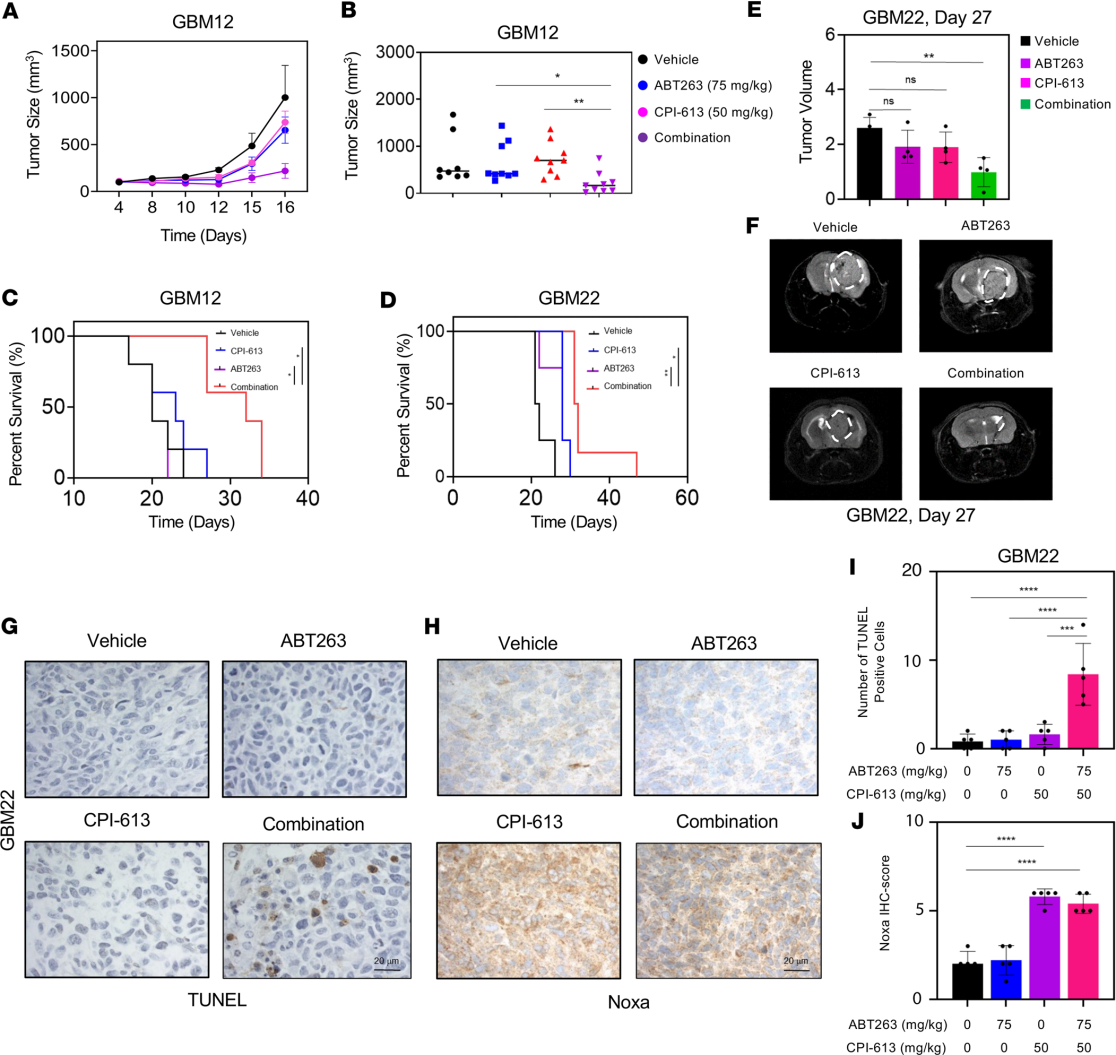
Dual inhibition of Bcl-xL and OGDH extends animal survival in orthotopic patient-derived xenograft models of GBM in mice. (**A** and **B**) GBM12 cells were implanted into the subcutis of immunocompromised *nu/nu* mice. Seven days later, the mice were divided into 4 treatment groups: vehicle, CPI-613 (50 mg/kg), ABT263 (75 mg/kg), and the combination of both. The tumor volume over time is shown on the left and the tumor volume on the last day of the experiment is shown on the right (*n* = 9 per group). (**C** and **D**) GBM12 and GBM22 cells were implanted in the right striatum of *nu/nu* mice. Four groups were randomly assigned: vehicle, CPI-613, ABT263, and the combination of both. Seven days after the implantation, mice were treated 3 times per week and animal survival is provided (Kaplan-Meier curve). The log-rank test was used to assess statistical significance (GBM12: *n* = 5 per group; GBM22: *n* = 4 for vehicle, CPI-613, and ABT263, and *n* = 6 for CPI-613 + ABT263). Median survival in GBM12: 20 days for vehicle and ABT263, 23 days for CPI-613, and 32 days for CPI-613 + ABT263. Median survival in GBM22: 21.5 days for vehicle, 28 days for ABT263 and CPI-613, and 31.5 days for CPI-613 + ABT263. (**E** and **F**) Representative MRI images of brain tumors as well as their quantification from the experiment in **D** are shown (Bruker BioSpec, 9.4 Tesla). (**G** and **I**) Shown are representative images of TUNEL staining and the related quantification in **I**. (**H** and **J**) Shown are representative images of Noxa immunohistochemical staining and the related quantification in **J**. Scale bars: 20 μm. Statistical significance was assessed by using 1-way ANOVA with Dunnett’s multiple-comparison test (**B**–**E**, **I**, and **J**). Data are shown as mean ± SEM in **A** and mean ± SD in **B**–**D**, **I**, and **J**. *P < 0.05; **P < 0.01; ***P < 0.001; ****P < 0.0001.

Since CPI-613 and ABT263 have reached clinical testing (19–21), it was tempting to evaluate whether these 2 compounds would elicit a synergistic growth reduction in vivo as well. We employed dosages of compounds that showed no weight loss following treatment in mice (Supplemental Figure 9A). Next, we tested the efficacy of the drug combination treatment in a patient-derived xenograft (PDX) model (GBM12). We found that the combination treatment of ABT263 and CPI-613 reduced the tumor growth compared with single treatments or vehicle treatment (Figure 6, A and B). Due to the promise of the drug combination treatment in the subcutaneous GBM xenograft model, we decided to assess the efficacy of this drug combination treatment in PDX orthotopic mouse model systems. Animals that received the combination treatment of ABT263 and CPI-613 had a significantly longer overall survival compared with the vehicle or single-drug treatment groups in 2 GBM PDX models, which implies potential clinical efficacy (Figure 6, C–F). H&E staining (Supplemental Figure 9B) and MRI imaging (Figure 6F) demonstrated a reduction in tumor growth following exposure to the combination treatment. With regard to the molecular changes, we found that CPI-613 increased the number of TUNEL-positive cells in orthotopic tumors of animals receiving the combination treatment of ABT263 and CPI-613 (Figure 6, G and I). Moreover, CPI-613 elicited an increase in the protein expression levels of Noxa in animals receiving either the single treatment (CPI-613) or the combination treatment (ABT263 + CPI-613) (Figure 6, H and J). Another critical feature of therapeutic interventions designed for brain tumors is their impact on neurons. To investigate this, we evaluated neuronal toxicity in vivo following the different drug treatments. We did not detect any form of neuronal cell death in the treatment groups (representative neurons from the hippocampal region are shown) (Supplemental Figure 9, C and D).

## Discussion

Metabolism and the microenvironment are currently key aspects that are being studied in GBM (24, 25) because these 2 features may be critical drivers of GBM growth and treatment resistance (26–28). The tumor microenvironment is centered on 2 main components, which is either the interaction of immune cells with tumor cells or neurons with GBM cells (29). It may even be conceivable that the 2 components are tightly interlinked. By far, myeloid-derived suppressor cells, regulatory T cells, and M2 macrophages are the main causes of an immunosuppressive environment in GBM that ultimately may allow GBM cells to evade killing by cytotoxic T cells.

Metabolism is probably the most critical component that dictates response and resistance since it affects both tumor cells and the microenvironment, and it has been recently suggested that metabolic reprogramming may be the driver of genetic changes that are responsible for tumor growth and ultimately treatment resistance (30). Here, through genetic screening, we have identified the *OGDH* gene as a critical driver of GBM growth. This was a surprising finding in that one would anticipate that the more proximal enzymes, such as citrate synthase, would be far more critical for GBM growth than the OGDH enzyme, especially in light of the fact that glutamine metabolism was found not to be as critical as glucose and acetate for GBM growth based on prior ^13^C tracing studies in brain tumor patients (31). In our model systems of human PDX GBM lines, we found that loss of OGDH was accompanied by a reduction in the OCR. This finding was anticipated since the OGDH enzyme is a key driver of the TCA cycle that, along with other enzymes in the cycle, generates NADH, which critically fuels the respiratory chain. In terms of translatability, we utilized an inhibitor of OGDH that has reached phase III clinical testing in patients, called CPI-613 (19–21). For the most part, this compound phenocopied our findings obtained in genetic loss-of-function experiments.

Loss of function of OGDH led to an activation of the ISR with an increase in ATF4 (Figure 4), which was preceded by a loss of ATP. Consequently, rescue experiments suggested that the activation of the ISR was related to loss of energy levels (Supplemental Figure 4E). While ATP did not completely restore viability upon inhibition of OGDH, the rescue was nevertheless statistically significant. However, these findings also suggest that alternate metabolites may be involved. In this regard, it is possible that certain amino acids may be involved as well, e.g., aspartate. Another explanation may be that the metabolite L-2-hydroxyglutarate (L-2-HG) may mediate parts of the antiglioma effects of CPI-613 or loss of function of OGDH. For instance, upon loss of OGDH in hematopoietic stem cells, L-2-HG accumulated and exerted inhibitory effects on oxidative energy metabolism (32).

We have found that following blockage of OGDH the transcription factor ATF4 was enriched in the promoter region of the *PMAIP1* gene, which encodes the proapoptotic Bcl-2 family member, Noxa. In turn, ATF4 increased Noxa levels. Through rescue experiments we found that the increase in Noxa was involved in the reduction in viability of GBM cells mediated by interference with OGDH, suggesting activation of classical intrinsic apoptosis (Figure 2). Because Noxa binds to Mcl-1 to facilitate the release of BAK from Mcl-1, high Noxa levels sensitize cancer cells to the cytotoxic effects of ABT263 and other BH3 mimetics (33). Indeed, we were able to demonstrate that inhibition of OGDH enhanced the efficacy of ABT263 in killing GBM cells (Figure 5). Based on silencing studies, these effects appeared to be mediated predominantly by loss of Bcl-xL. As anticipated, we noted that silencing of Noxa protected from ABT263-mediated reduction in the viability of GBM cells (Figure 5E). Our study establishes a functional link between loss of function of OGDH, ISR signaling, and intrinsic apoptosis. These results position OGDH as a treatment target for GBM. Our findings are distinct from previous results obtained in breast and colonic carcinoma models, which showed an increased dependency on OGDH only in the presence of mutated PIK3CA (17). While we cannot completely exclude the possibility that certain genetic changes may render GBM cells even more dependent on OGDH for survival, it seems more likely that OGDH is a key driver in a broad range of IDH-wild-type GBM likely to be independent of specific genetic mutations, such as PIK3CA. We created 2 orthotopic GBM models to demonstrate that the combination treatment of ABT263 and CPI-613 is efficacious in vivo. In both model systems, the combination treatment led to an increase in animal survival, which was more pronounced with the GBM12 cells. The difference in response to therapy between the GBM12 and GBM22 cells may potentially be explained by the different behavior of these cells when exposed to the brain parenchyma. However, other explanations may be possible as well. We also have not formally determined the extent of blood-brain barrier penetration of both ABT263 and CPI-613. While we see an antiglioma effect in our models (including orthotopic models), it is possible that the efficacy of the drug combination may be mitigated due to the potential limited delivery of the compounds. A recent study suggested that some GBMs may be characterized as a mitochondrial subtype since these tumors appear to be particularly reliant on cellular respiration and oxidative phosphorylation (34). In agreement, classical inhibitors of either cellular respiration, e.g., metformin or the more specific complex I inhibitor, IACS-010759, or blockers of mitochondrial protein translation (tigecycline) reduced the viability of “mitochondrial subtype” GBM cells more potently (34). Based on these findings, it appears likely that CPI-613 may be efficacious in this GBM subtype as well.

While our studies focused exclusively on tumor cell metabolism, it remains to be determined how OGDH affects the immune microenvironment in GBM. This is of relevance since immunosuppressive cell types rely on oxidative energy metabolism. Therefore, it may be conceivable that myeloid-derived suppressor cells display a dependency on OGDH to exert their immunosuppressive effects in GBM. All in all, we have established OGDH as a target for GBM, which significantly extends our current understanding about GBM metabolism and how OGDH affects ISR signaling as well as intrinsic apoptosis.

## Methods

### Sex as a biological variable.

Sex was not regarded as a biological variable in this study. Our assessment focused solely on female mice, examining the effects of vehicle, single, and combination drug treatments.

### Cell cultures.

KNS42, GBM22, GBM43, and GBM12 GBM cells were cultured in DMEM (Thermo Fisher Scientific, MT10013CV), 10% FBS (Gemini), and 100 μg/mL Primocin (Invivogen, ant-pm-1). KNS42 cells were purchased from the Japanese Collection of Research Bioresources Cell Bank (JCRB, IFO50356). GBM22, GBM43, GBM12 cells were obtained from Jann Sarkaria (Mayo Clinic, Rochester, Minnesota, USA) between 2020 and 2023. For the treatment experiments, cells were cultured in DMEM containing 1.5% FBS and 100 μg/mL Primocin. NCH644 stem cell–like glioma cells were purchased from Cell Line Services (catalog 820403) and were cultured in StemPro NSC SFM (Thermo Fisher Scientific, A1050901) with 100 μg/mL Primocin for maintenance and for drug treatment. All cells were incubated and maintained at 37°C and 5% CO_2_.

### Reagents.

CPI-613 (devimistat; HY-15453), navitoclax (ABT-263; HY-10087), A-1210477 (HY-12468), and venetoclax (ABT-199; HY-15531) were purchased from MedChemExpress. Z-VAD-FMK (S7023) and ATP disodium (S1985) were purchased from Selleckchem. Puromycin dihydrochloride (P9620) was purchased from Sigma-Aldrich. A 10 mM working solution in dimethylsulfoxide (DMSO) was prepared for all reagents, with a final DMSO concentration below 0.1% (v/v).

### Cell viability assays.

Cells were seeded at a density of 3,000 (KNS42, GBM22, and GBM43) or 8,000 cells (GBM12 and NCH644) per well in a 96-well plate and allowed to attach overnight. The next day, cells were treated with targeted drugs for 72 hours and were analyzed for cell viability by using the CellTiter-Glo assay (Promega, G7571). For the ATP rescue experiments, cell viability was determined by using a CyQUANT Cell Proliferation Assay (Thermo Fisher Scientific, C7026). To evaluate drug synergy, the median effect equation (Chou-Talalay) was used. Following calculations, this approach yields normalized isobolograms and the combination index, respectively (35).

### Flow cytometry.

Cells were seeded at a density of 30,000 (KNS42, GBM22, and GBM43) or 80,000 cells (GBM12 and NCH644) per well in a 12-well plate and allowed to attach overnight. On the day of the experiment, the cells were stained with FITC Annexin V/PI (BD Biosciences, 556420) to detect apoptosis and necrosis following the company’s instructions. Samples were detected by using LSRII flow cytometry (BD) and the data were analyzed with FlowJo software (version 8.7.1, Tree Star).

### RNA-seq and subsequent GSEA.

KNS42 cells were treated with DMSO or 100 μM CPI-613 for 24 hours. Cells were extracted for total RNA by using the miRNeasy Mini Kit (QIAGEN, 217004). RNA (100 ng) with RIN greater than 8 was mixed with the RNA library SIRV-Set 1 (Iso Mix E0, E1, E2) - RNA-seq (Lexogene, SKU 025.03) according to the company’s instructions. The RNA-seq was performed at the Columbia Genome Center and the GSEA was performed by using Scidap (https://scidap.com/).

### Western blotting and protein capillary electrophoresis.

All samples were lysed in 1× Laemmli buffer (Bio-Rad) containing protease and phosphatase inhibitor cocktail (Thermo Fisher Scientific, 78440). For standard Western blotting, samples were run in a 4%–12% SDS-PAGE gel (Invitrogen, NP0321BOX) and were transferred to Immun-Blot PVDF membranes (Bio-Rad, 1620177). The blots were captured by using the Azure c300 imaging system (Azure Biosystems). For protein capillary electrophoresis, samples were detected by using the Wes instrument (ProteinSimple) following the company’s instructions.

Primary antibodies against the following proteins were used in standard Western blotting at the indicated dilutions: Mcl-1 (clone D35A5) (Cell Signaling Technology [CST], catalog 5453; 1:500), Bcl-xL (clone 54H6) (CST, catalog 2764; 1:500), Bcl-2 (clone D55G8) (CST, catalog 4223; 1:500), Noxa (Calbiochem, catalog OP180, clone 114C307; 1:500), β-actin (Sigma-Aldrich, catalog A1978, clone AC15; 1:3,000), ATF3 (clone D2Y5W) (CST, catalog 33593; 1:500), ATF4 (clone D4B8) (CST, catalog 11815; 1:500), PARP (clone 46D11) (CST, catalog 9532; 1:500), cleaved caspase-9 (Asp330) (clone D2D4) (CST, catalog 7237; 1:500), and caspase-3 (clone 8G10) (CST, catalog 9665; 1:500). The following secondary antibodies were used in the standard Western blot: anti–rabbit IgG (H+L), HRP (Thermo Fisher Scientific, 31460) and anti–mouse IgG (H+L), HRP (Thermo Fisher Scientific, 31430).

Primary antibodies against the following proteins were used in the protein capillary electrophoresis: vinculin (Abcam, catalog ab129002; 1:500), AMPK (clone D5A2) (CST, catalog 5831; 1:25), p-AMPK (Thr172) (CST, catalog 2531; 1:25), and ATF4 (clone D4B8) (CST, catalog 11815; 1:25). The secondary antibodies were anti-rabbit–HRP (ProteinSimple, 042-206) and anti-mouse–HRP (ProteinSimple, 042-205).

### Real-time PCR analysis.

Cells were extracted for total RNA by using the miRNeasy Mini Kit. The RNA was synthesized to cDNA by using the qScript cDNA SuperMix (QuantaBio kit, 101414-106). The RT-PCR was performed by using PerfeCTa SYBR Green FastMix Reaction Mixes (Quantabio, 101414-276) on a qPCR instrument (Quantabio) with the following steps: 95°C for 10 minutes, followed by 40 cycles of 95°C for 15 seconds, 60°C for 30 seconds, and 72°C for 30 seconds. All RT-PCR was performed in quadruplicate and the average fold changes were calculated based on *18S* in the threshold cycle (Cq). Primer sequences are provided in Supplemental Table 1.

### siRNA transfection and lentiviral transduction.

The following siRNAs were purchased from Dharmacon: nontargeting siRNA-pool (scramble; D-001810–10–20), ATF4 siRNA-pool (L-005125-00-0005), ATF4-10 siRNA-10 (J-005125-10-0002), ATF4-12 siRNA-12 (J-005125-12-0002), ATF3 siRNA-pool (L-008663-00-0005), OGDH siRNA-pool (L-009679-00-0005), Mcl1-16 siRNA (J-004501-16-0002), and Mcl1-17 siRNA (J-004501-17-0002). PMAIP1-1 siRNA (s10708) and PMAIP1-2 siRNA (s10709) were purchased from Thermo Fisher Scientific.

Cells were transfected with Bcl-xL-1 (CST, 6362) and Bcl-xL-2 (CST, 6363) siRNA using Lipofectamine RNAiMAX (Invitrogen, 13778075) according to the manufacturers’ instructions.

OGDH shRNAs (TRCN0000028618, TRCN0000028643, and TRCN0000028580) were purchased from Sigma-Aldrich. Adeno CMV Null Adenovirus (Ad-CMV-Null, 1300) and human BCL2L1 Adenovirus (ADV-202038) were purchased from Vector Biolabs. Lentivirus was generated by transfection of pMD2.G, psPAX2, and the relevant lentivirus plasmid into 293T cells for 72 hours. The viral supernatant was collected, filtered with a 0.45-μm surfactant-free cellulose acetate (SFCA) syringe filter (Thermo Fisher Scientific, 09-754-21), and concentrated with an Amicon Ultra-15 centrifugal filter unit (Sigma-Aldrich, UFC910024) before they were transduced into GBM cells. GBM cells were transduced with lentivirus for 48 hours and were selected with puromycin (2 μg/mL) for 1 week.

### Isotope tracing and LC/MS.

Cells were seeded at a density of 1 × 10^6^ cells per 10 cm dish and allowed to attach overnight. The next day, cells were exposed to DMEM without glucose, glutamine, or phenol red (Thermo Fisher Scientific, A1443001), containing 25 mM (U-^13^C_6_) D-glucose (Cambridge Isotope Laboratories, Inc, CLM-1396–2), 4 mmol/L glutamine (Thermo Fisher Scientific, 15410314), and 1.5% dialyzed FBS (Thermo Fisher Scientific, A3382001) for 24 hours. Cells were collected and analyzed for polar metabolites by the Metabolomics Core Facility at Weill Cornell.

### Extracellular flux analysis.

Cells were seeded at a density of 3 × 10^4^ cells per XFe24 cell culture microplate (Agilent) and allowed to attach overnight. Cells were treated with target drugs for 24 hours. The OCR was measured with a Seahorse XFe24 analyzer (Agilent) using the Mito Stress Assay kit (Agilent, 103015–100) in Seahorse XF base medium (Agilent, 102353–100) containing 10 mM glucose, 2 mM glutamine, and 1 mM pyruvate. The following compounds were injected in sequential order: 2 μM oligomycin, 2 μM carbonyl cyanide-4 (trifluoromethoxy) phenylhydrazone, and 0.5 μM rotenone/antimycin, following the manufacturer’s instructions (Agilent).

### Subcutaneous xenograft and orthotopic GBM PDX mouse model.

Subcutaneous xenograft: 3 × 10^6^ GBM12 cells were implanted subcutaneously into the flanks of 6- to 8-week-old female nude mice (Taconic Biosciences, NCRNU-F sp/sp, CrTac:NCr-*Foxn1^nu^*). ABT263 (75 mg/kg) and CPI-613 (50 mg/kg) were dissolved in a mixture containing DMSO, cremophor EL (Sigma-Aldrich, 61791–12–6), ethyl alcohol (Pharmco-Aaper, 200 proof), and PBS at a ratio of 10:32:8:50 (v/v/v/v). Intraperitoneal treatment was administered 3 times per week. Tumor sizes were measured with a caliper and were calculated as (length × width^2^)/2.

Orthotopic PDX models: 5 × 10^4^ GBM12 and GBM22 cells were intracranially injected at 3 mm lateral of the bregma and 3 mm down in 6- to 8-week-old female nude mice. Treatments were given until the animals became moribund or when neurologic deficits were observed (retardation, lethargy, seizures). The dosages for ABT263 and CPI-613 were based on our earlier published work (7, 22, 36). The survival curve was analyzed by Kaplan-Meier survival fractions and the log-rank test was employed to assess the statistical significance. Immunohistochemical analyses for TUNEL and Noxa were performed as previously described (37). Regarding the quantification, a Noxa immunohistochemical score was assigned by taking into account both the staining intensity (0, no staining; 1, weak staining intensity; 2, intermediate staining intensity; 3, strong staining intensity) and the percentage of positive cells (0, no cells labeled; 1, 1%–25% cells labeled; 2, 25%–74% cells labeled; 3, 75%–100% cells labeled).

### Statistics.

Statistical significance was determined by using Prism version 9 (GraphPad). The 2-tailed Student’s *t* test or ANOVA (for multiple comparisons) was used. A *P* value of less than 0.05 was considered statistically significant.

### Study approval.

All procedures were performed in accordance with Animal Welfare Regulations and approved by the Institutional Animal Care and Use Committee at the Columbia University Medical Center (AC-AABC6505 and AC-AABI3633).

### Data availability.

All data are included in the Supporting Data Values file. Any data that support the findings of this study are available from the corresponding author upon reasonable request. The RNA-seq data used in this study were deposited in the NCBI Gene Expression Omnibus database (GEO GSE223297; https://www.ncbi.nlm.nih.gov/geo/query/acc.cgi?acc=GSE223297).

## Author contributions

TTTN, CT, and MDS conceived and designed the study. TTTN, CT, ES, CS, JYM, and QG developed the methodology. TTTN, CT, ES, CS, NH, GZ, and MDS acquired data. TTTN, GKM, MAW, and MDS wrote, reviewed, and/or revised the manuscript. HOA, JNB, and PC provided material support. MDS supervised the study.

## Supplementary Material

Supplemental data

Unedited blot and gel images

Supporting data values

## Version 1 03/14/2024

In-Press Preview

## Version 2 04/22/2024

Electronic publication
