# Supplementary material for: OGDH and Bcl-xL loss causes synthetic lethality in glioblastoma
Source: JCI Insight. 2024 Mar 14;9(8):e172565. doi: 10.1172/jci.insight.172565 (PMC11141877; doi:10.1172/jci.insight.172565)
Supplement: Supplemental data [file jciinsight-9-172565-s118.pdf]

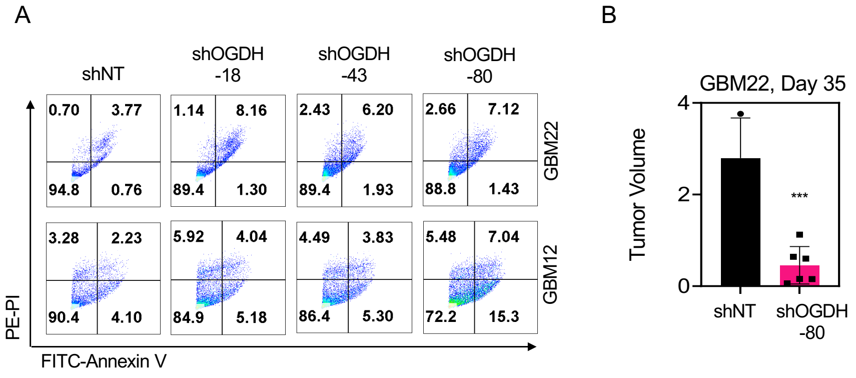
A
shNT
shOGDH -18
shOGDH -43
shOGDH -80
0.70
3.77
94.8
0.76
1.14
8.16
89.4
1.30
2.43
6.20
89.4
1.93
2.66
7.12
88.8
1.43
GBM22
3.28
2.23
90.4
4.10
5.92
4.04
84.9
5.18
4.49
3.83
86.4
5.30
5.48
7.04
72.2
15.3
GBM12
PE-PI
FITC-Annexin V
B
GBM22, Day 35
Tumor Volume
4
2
0
***
shNT
shOGDH -80

Supplementary Figure 1. Loss of OGDH induces apoptosis in GBM cells. (A) GBM22 and GBM12 cells were transduced with lentiviruses encoding either a non-targeting shRNA (shNT) or shRNAs against OGDH. Following establishment of the cells, the transduced cells were subjected to Annexin V/ PI staining to detect apoptotic cells. (**B)** GBM22 cells were transduced with lentiviral vectors, containing either non targeting shRNA or shRNA against OGDH, and were implanted in the right striatum of nude mice. Shown is the tumor size of GBM22-shNT and GBM22-shOGDH-80. Statistical significance was assessed by two-tailed Student t test in B. Data are shown as mean ± SD. *** $p < 0.001$.

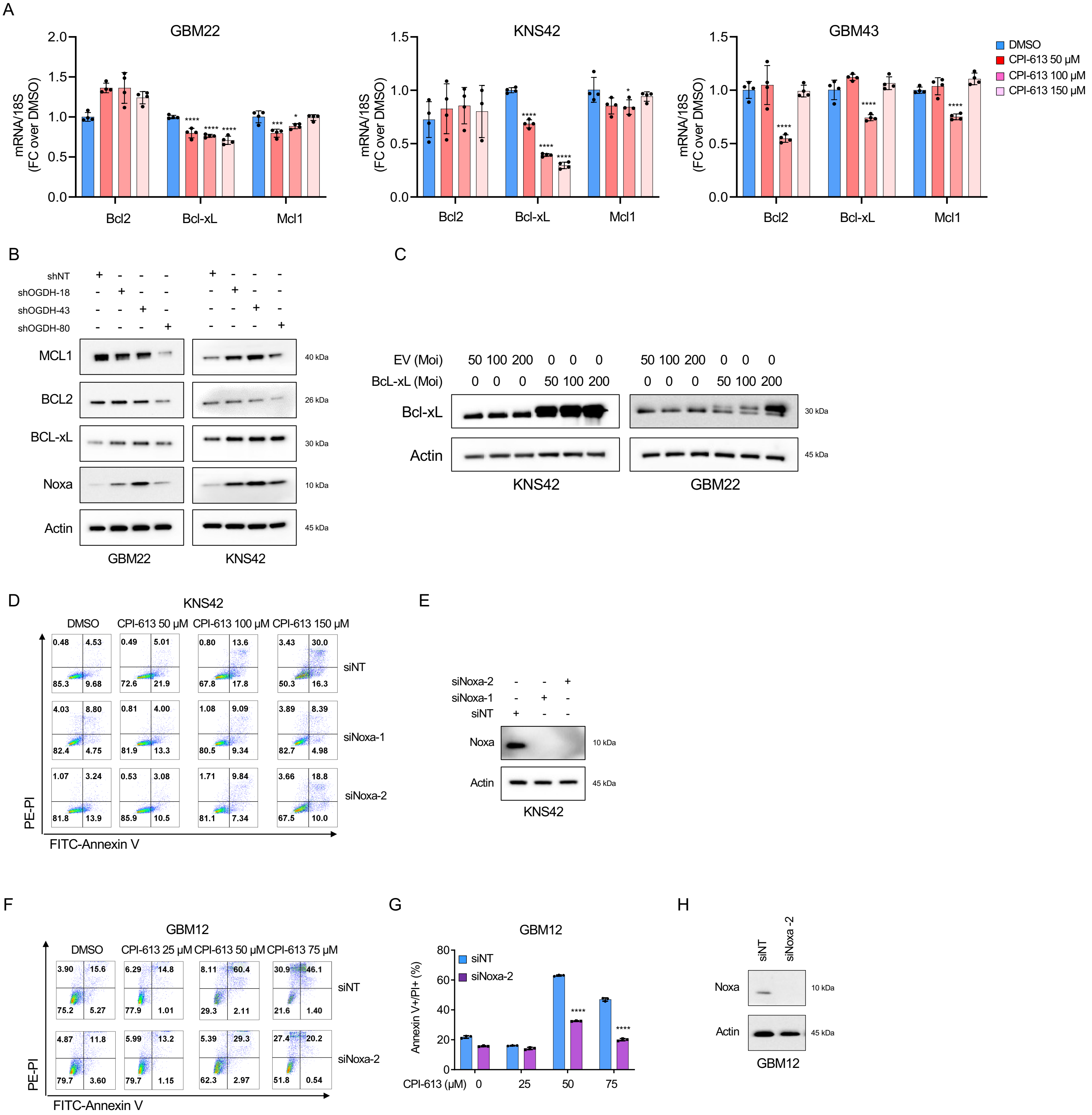

A
GBM22
KNS42
GBM43
mRNA/18S (FC over DMSO)
Bcl2
Bcl-xL
Mcl1
DMSO
CPI-613 50 μM
CPI-613 100 μM
CPI-613 150 μM
B
shNT
shOGDH-18
shOGDH-43
shOGDH-80
MCL1
BCL2
BCL-xL
Noxa
Actin
40 kDa
26 kDa
30 kDa
10 kDa
45 kDa
GBM22
KNS42
C
EV (Moi) 50 100 200 0 0 0
BcL-xL (Moi) 0 0 0 50 100 200
Bcl-xL
Actin
30 kDa
45 kDa
KNS42
GBM22
D
KNS42
DMSO
CPI-613 50 μM
CPI-613 100 μM
CPI-613 150 μM
siNT
siNoxa-1
siNoxa-2
PE-PI
FITC-Annexin V
E
siNoxa-2
siNoxa-1
siNT
Noxa
Actin
10 kDa
45 kDa
KNS42
F
GBM12
DMSO
CPI-613 25 μM
CPI-613 50 μM
CPI-613 75 μM
siNT
siNoxa-2
PE-PI
FITC-Annexin V
G
GBM12
siNT
siNoxa-2
Annexin V+/PI+ (%)
CPI-613 (μM) 0 25 50 75
H
siNT
siNoxa -2
Noxa
Actin
10 kDa
45 kDa
GBM12

Supplementary Figure 2. The role of the Bcl-2 family members of proteins in CPI-613 mediated loss of viability. (A) Real time PCR analysis of GBM22, KNS42, and GBM43 cells treated with increasing concentrations of CPI-613 for 24 hours. 18S is a housekeeping gene (n = 4 per group). (**B)** Western blots of the Bcl-2 family of proteins obtained from GBM22 and KNS42 cells transduced with non-targeting shRNA (shNT) or with shRNAs against OGDH. (**C**) Western blots of GBM22 and KNS42 cells that were transduced with an empty vector (EV) or a vector containing Bcl-xL cDNA (using adenoviruses). (**D)** KNS42 cells were transfected with non-specific siRNA (siNT) or with siRNAs against Noxa, were treated with increasing concentrations of CPI-613, stained with Annexin V/PI and analyzed by flow cytometry (n=3 per group). (**E)** Shown are Noxa and Actin western blots of KNS42 cells transfected with non-targeting siRNA (siNT) or with Noxa specific siRNAs. Actin is a loading control. (**F** and **G)** GBM12 cells were transfected with non-targeting siRNA or with siRNA against Noxa, were treated with increasing concentrations of CPI-613, stained with Annexin V/PI and analyzed by flow cytometry (n=3 per group). Quantification is shown in **G**. (**H)** Shown are Noxa and actin western blots of GBM12 cells transfected with non-targeting siRNA (siNT) or with a siRNA against Noxa. Statistical significance was assessed ANOVA with Dunnett multiple comparison test in A and by two-tailed Student t test in G. Data are shown as mean ± SD. $^*p < 0.05$, ***/**** $p < 0.001$.

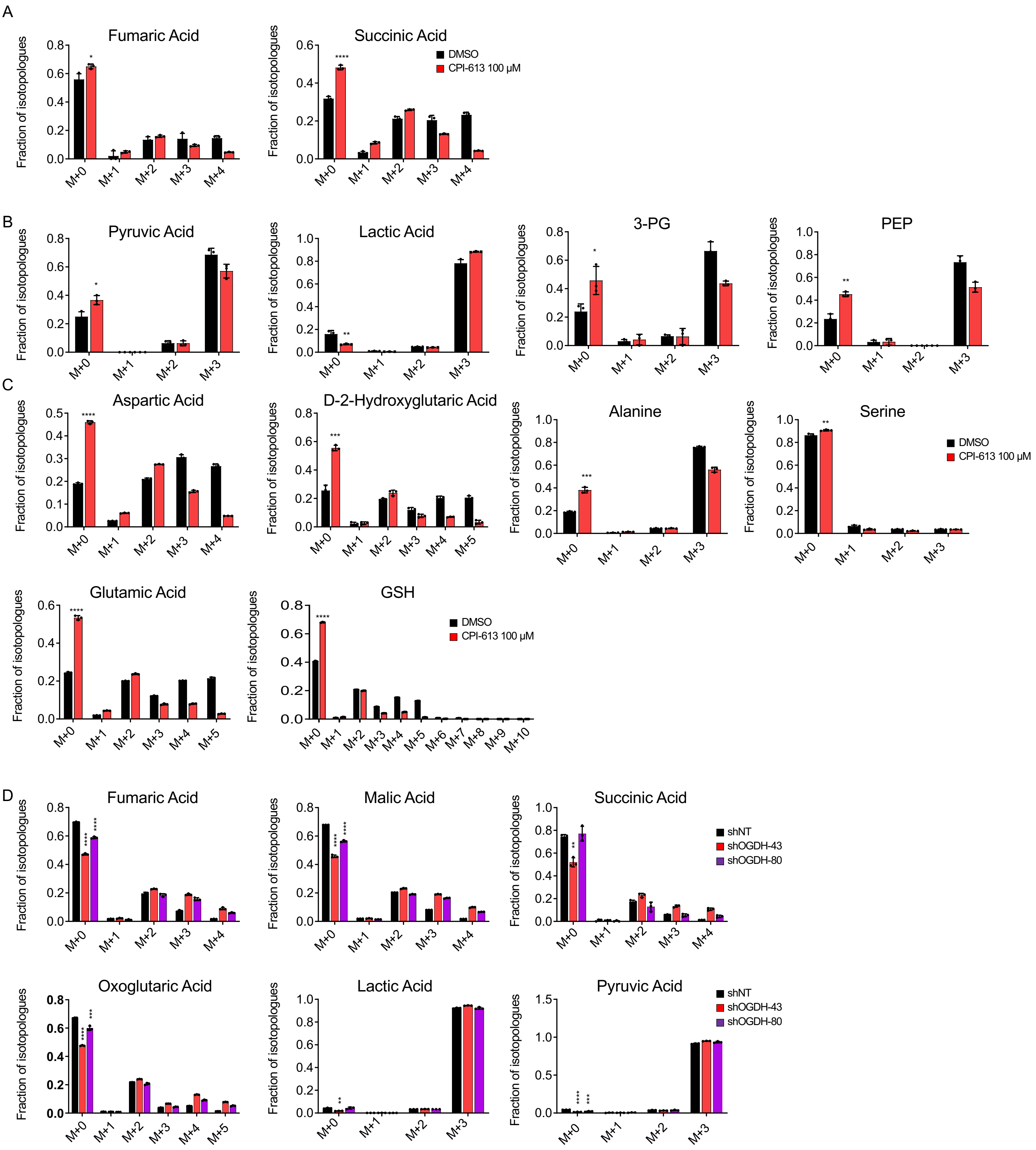

A
Fumaric Acid
Succinic Acid
DMSO
CPI-613 100 μM
Fraction of isotopologues
M+0
M+1
M+2
M+3
M+4
B
Pyruvic Acid
Lactic Acid
3-PG
PEP
C
Aspartic Acid
D-2-Hydroxyglutaric Acid
Alanine
Serine
Glutamic Acid
GSH
M+5
M+6
M+7
M+8
M+9
M+10
D
Fumaric Acid
Malic Acid
Succinic Acid
shNT
shOGDH-43
shOGDH-80
Oxoglutaric Acid
Lactic Acid
Pyruvic Acid

Supplementary Figure 3. OGDH regulates glucose carbon flux into the TCA cycle. (A-C) KNS42 cells were treated with 100 μM CPI-613 in the DMEM media containing 25 mM U-13C glucose, 4 mM glutamine and 1.5% dialyzed FBS for 24 hours. Shown is the fraction of the isotopologues for each metabolite (n = 3 per group). (**D)** GBM22 cells were transduced with lentiviral vectors, containing either non-targeting shRNA or shRNAs against OGDH. The transduced cells were cultured in the DMEM media containing 25 mM U-13C glucose, 4 mM glutamine and 10% dialyzed FBS for 24 hours. Shown is the fraction of the isotopologues for each metabolite (n = 3 per group). Statistical significance was assessed by two-tailed Student t test in A-D. Data are shown as mean ± SD. *p < 0.05, ***/**** p < 0.001.

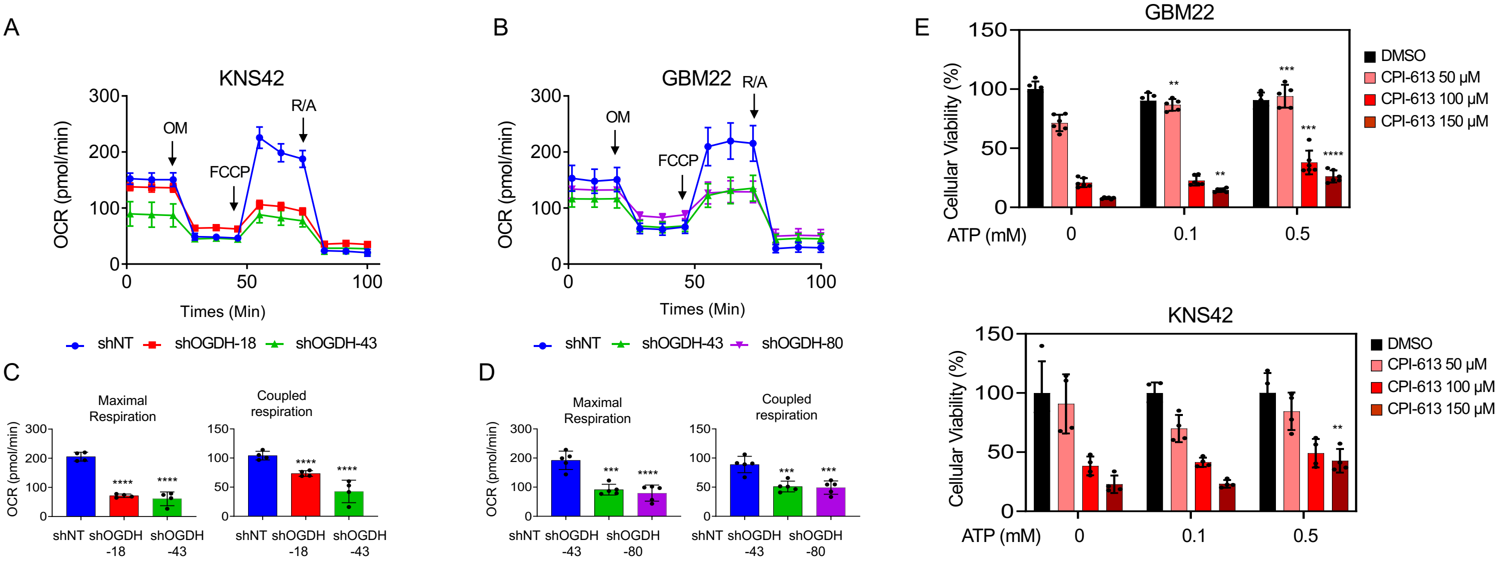
A
KNS42
OCR (pmol/min)
OM
FCCP
R/A
Times (Min)
shNT
shOGDH-18
shOGDH-43
B
GBM22
OCR (pmol/min)
OM
FCCP
R/A
Times (Min)
shNT
shOGDH-43
shOGDH-80
C
Maximal Respiration
Coupled respiration
OCR (pmol/min)
shNT
shOGDH -18
shOGDH -43
D
Maximal Respiration
Coupled respiration
OCR (pmol/min)
shNT
shOGDH -43
shOGDH -80
E
GBM22
Cellular Viability (%)
ATP (mM)
DMSO
CPI-613 50 μM
CPI-613 100 μM
CPI-613 150 μM
KNS42
Cellular Viability (%)
ATP (mM)
DMSO
CPI-613 50 μM
CPI-613 100 μM
CPI-613 150 μM

Supplementary Figure 4. Loss of function of OGDH reduces the oxygen consumption rate in GBM cells. (**A-D)** KNS42 and GBM22 cells were transduced with either non targeting shRNA or shRNAs against OGDH. The transduced cells were subjected to extracellular flux analysis to analyze maximal respiration and coupled respiration in **C** and **D** (n=5 per group). (**E)** GBM22 and KNS42 cells were treated with CPI-613 for 24 hours in the presence or absence of ATP and cell viability was analyzed (n=4 per group). Statistical significance was assessed ANOVA with Dunnett multiple comparison test in C and D and by two-tailed Student t test in E. Data are shown as mean ± SD. $^{**}p < 0.01$, ***/**** $p < 0.001$.

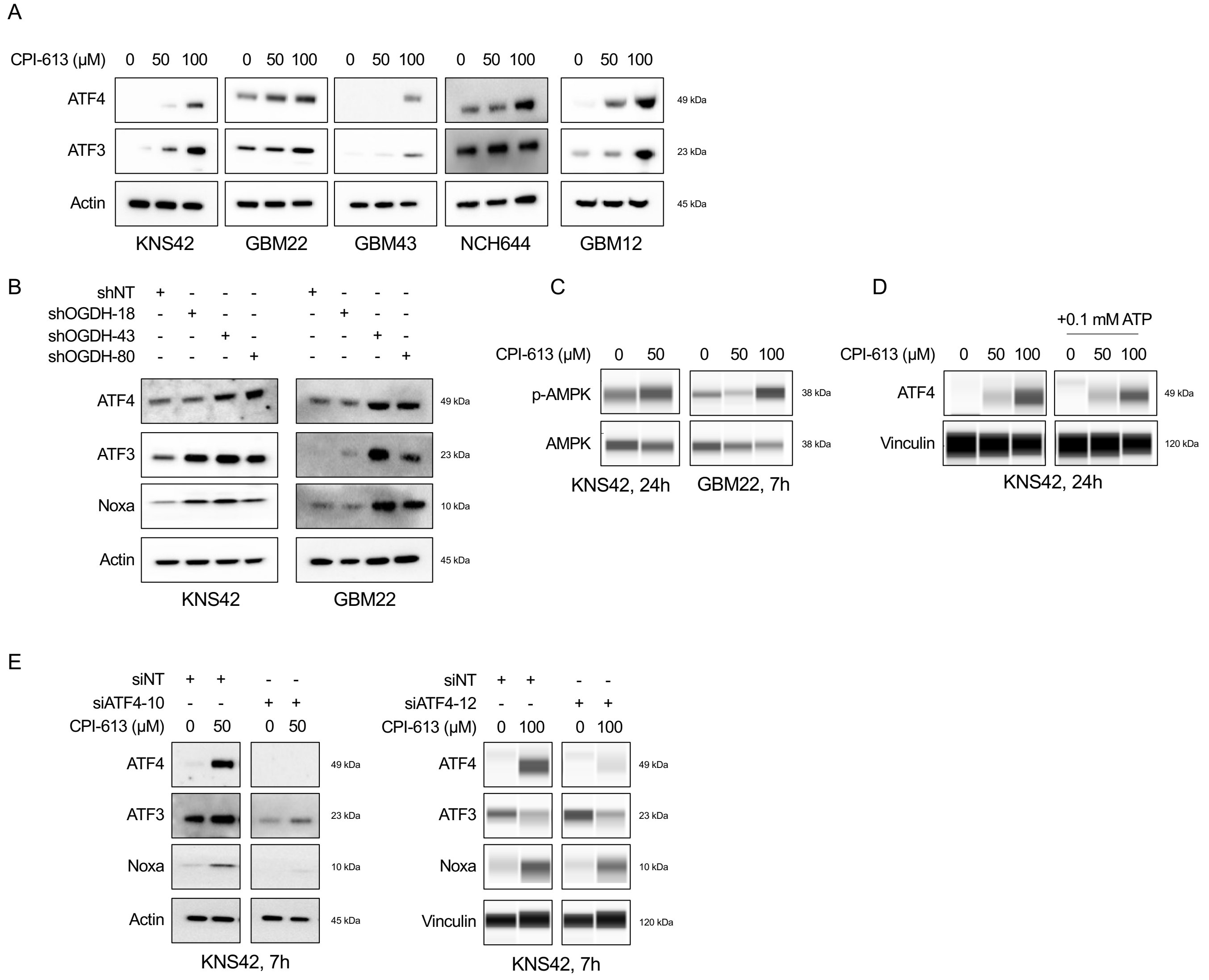

A
CPI-613 (μM) 0 50 100 0 50 100 0 50 100 0 50 100 0 50 100
ATF4
ATF3
Actin
49 kDa
23 kDa
45 kDa
KNS42
GBM22
GBM43
NCH644
GBM12
B
shNT + - - - + - - -
shOGDH-18 - + - - - + - -
shOGDH-43 - - + - - - + -
shOGDH-80 - - - + - - - +
ATF4
ATF3
Noxa
Actin
49 kDa
23 kDa
10 kDa
45 kDa
KNS42
GBM22
C
CPI-613 (μM) 0 50 0 50 100
p-AMPK
AMPK
38 kDa
38 kDa
KNS42, 24h
GBM22, 7h
D
+0.1 mM ATP
CPI-613 (μM) 0 50 100 0 50 100
ATF4
Vinculin
49 kDa
120 kDa
KNS42, 24h
E
siNT + + - -
siATF4-10 - - + +
CPI-613 (μM) 0 50 0 50
ATF4
ATF3
Noxa
Actin
49 kDa
23 kDa
10 kDa
45 kDa
KNS42, 7h
siNT + + - -
siATF4-12 - - + +
CPI-613 (μM) 0 100 0 100
ATF4
ATF3
Noxa
Vinculin
49 kDa
23 kDa
10 kDa
120 kDa
KNS42, 7h

Supplementary Figure 5. Loss of function of OGDH triggers the integrated stress response in GBM cells. (A) ATF3, ATF4 and Actin western blots of KNS42, GBM22, NCH644, GBM43, and GBM12 cells treated with increasing concentrations of CPI-613 for 24 hours. Actin is a loading control. (**B)** Western blots of GBM22 and KNS42 cells transduced with either non targeting shRNA or shRNAs against OGDH. (**C)** Shown are protein capillary electrophoresis analyses of KNS42 and GBM22 cells. (**D)** Shown are protein capillary electrophoresis analyses of KNS42 cells treated with CPI-613 in the presence or absence of ATP for 24 hours. (**E)** ATF3, ATF4, Noxa and Actin standard western blots (left) or protein capillary electrophoresis (right) analyses of KNS42 cells transfected with non-targeting siRNA (siNT) or with an siRNA against ATF4 (single siRNA) and treated with CPI-613 for 7h.

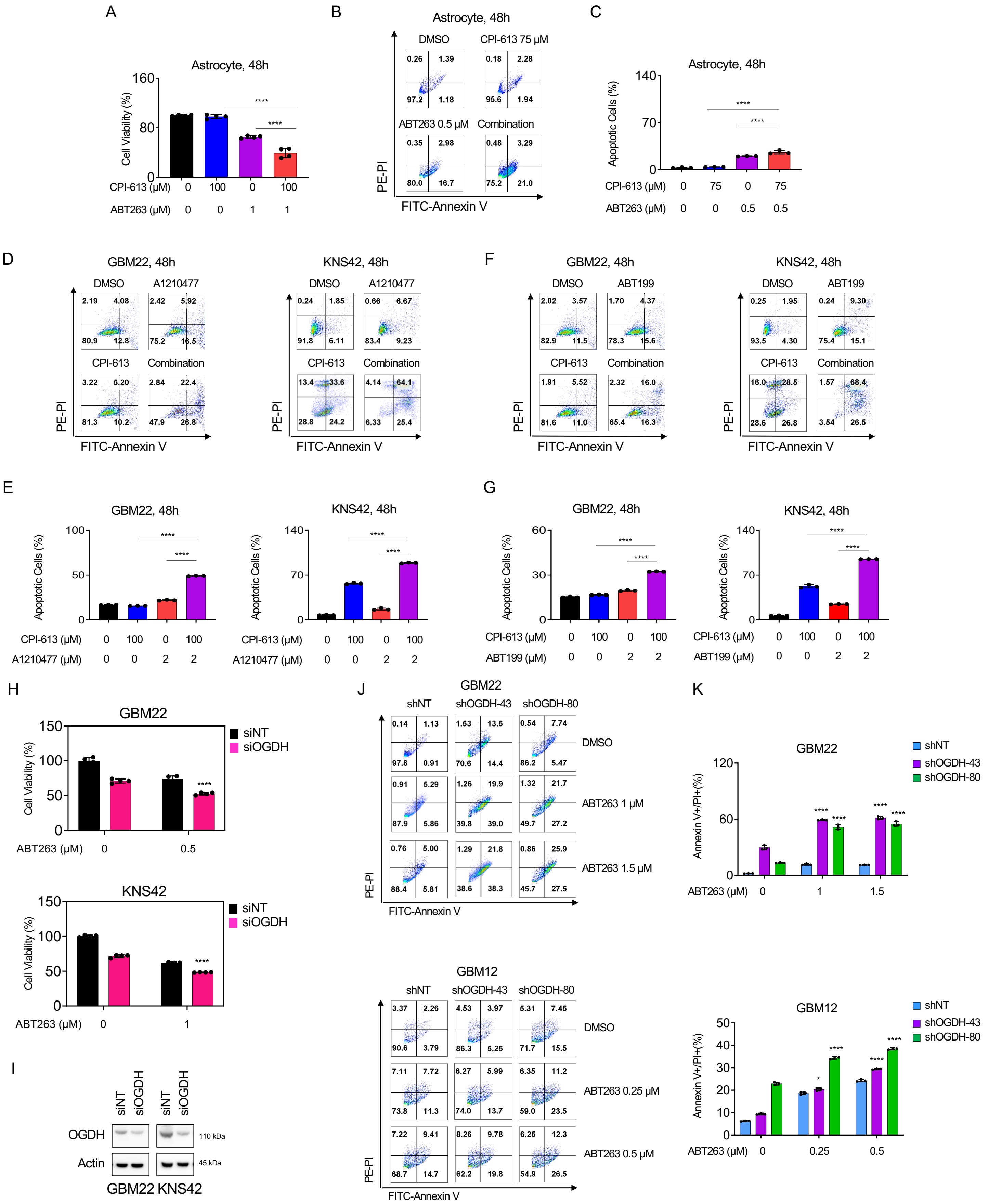

A
Astrocyte, 48h
Cell Viability (%)
160
80
0
****
****
CPI-613 (μM) 0 100 0 100
ABT263 (μM) 0 0 1 1
B
Astrocyte, 48h
DMSO
CPI-613 75 μM
0.26 1.39
97.2 1.18
0.18 2.28
95.6 1.94
ABT263 0.5 μM
Combination
0.35 2.98
80.0 16.7
0.48 3.29
75.2 21.0
PE-PI
FITC-Annexin V
C
Astrocyte, 48h
Apoptotic Cells (%)
140
70
0
****
****
CPI-613 (μM) 0 75 0 75
ABT263 (μM) 0 0 0.5 0.5
D
GBM22, 48h
DMSO
A1210477
2.19 4.08
80.9 12.8
2.42 5.92
75.2 16.5
CPI-613
Combination
3.22 5.20
81.3 10.2
2.84 22.4
47.9 26.8
PE-PI
FITC-Annexin V
KNS42, 48h
DMSO
A1210477
0.24 1.85
91.8 6.11
0.66 6.67
83.4 9.23
CPI-613
Combination
13.4 33.6
28.8 24.2
4.14 64.1
6.33 25.4
PE-PI
FITC-Annexin V
F
GBM22, 48h
DMSO
ABT199
2.02 3.57
82.9 11.5
1.70 4.37
78.3 15.6
CPI-613
Combination
1.91 5.52
81.6 11.0
2.32 16.0
65.4 16.3
PE-PI
FITC-Annexin V
KNS42, 48h
DMSO
ABT199
0.25 1.95
93.5 4.30
0.24 9.30
75.4 15.1
CPI-613
Combination
16.0 28.5
28.6 26.8
1.57 68.4
3.54 26.5
PE-PI
FITC-Annexin V
E
GBM22, 48h
Apoptotic Cells (%)
100
50
0
****
****
CPI-613 (μM) 0 100 0 100
A1210477 (μM) 0 0 2 2
KNS42, 48h
Apoptotic Cells (%)
140
70
0
****
****
CPI-613 (μM) 0 100 0 100
A1210477 (μM) 0 0 2 2
G
GBM22, 48h
Apoptotic Cells (%)
60
30
0
****
****
CPI-613 (μM) 0 100 0 100
ABT199 (μM) 0 0 2 2
KNS42, 48h
Apoptotic Cells (%)
140
70
0
****
****
CPI-613 (μM) 0 100 0 100
ABT199 (μM) 0 0 2 2
H
GBM22
Cell Viability (%)
150
100
50
0
siNT
siOGDH
****
ABT263 (μM) 0 0.5
KNS42
Cell Viability (%)
150
100
50
0
siNT
siOGDH
****
ABT263 (μM) 0 1
I
siNT siOGDH siNT siOGDH
OGDH
110 kDa
Actin
45 kDa
GBM22 KNS42
J
GBM22
shNT
shOGDH-43
shOGDH-80
0.14 1.13
97.8 0.91
1.53 13.5
70.6 14.4
0.54 7.74
86.2 5.47
DMSO
0.91 5.29
87.9 5.86
1.26 19.9
39.8 39.0
1.32 21.7
49.7 27.2
ABT263 1 μM
0.76 5.00
88.4 5.81
1.29 21.8
38.6 38.3
0.86 25.9
45.7 27.5
ABT263 1.5 μM
PE-PI
FITC-Annexin V
GBM12
shNT
shOGDH-43
shOGDH-80
3.37 2.26
90.6 3.79
4.53 3.97
86.3 5.25
5.31 7.45
71.7 15.5
DMSO
7.11 7.72
73.8 11.3
6.27 5.99
74.0 13.7
6.35 11.2
59.0 23.5
ABT263 0.25 μM
7.22 9.41
68.7 14.7
8.26 9.78
62.2 19.8
6.25 12.3
54.9 26.5
ABT263 0.5 μM
PE-PI
FITC-Annexin V
K
GBM22
shNT
shOGDH-43
shOGDH-80
Annexin V+/PI+(%)
120
60
0
**** ****
**** ****
ABT263 (μM) 0 1 1.5
GBM12
shNT
shOGDH-43
shOGDH-80
Annexin V+/PI+(%)
50
40
30
20
10
0
* ****
**** ****
ABT263 (μM) 0 0.25 0.5

Supplementary Figure 6. Inhibition of OGDH sensitizes for BH3-mimetic mediated cell death in GBM cells. (A) Astrocyte cells were treated with 100 μM CPI-613, 1 μM ABT263, or the combination of both for 72 hours, and were analyzed by a cell viability assay (n=4 per group). (**B** and **C)** Astrocyte cells were treated with 75 μM CPI-613, 0.5 μM ABT263, or the combination of both for 48 hours, were subjected to the Annexin V/ PI staining and analyzed by flow cytometry. The quantification is shown in **C** (n=3 per group). (**D** and **E)** GBM22 and KNS42 cells were treated with CPI-613, A1210477, or the combination of both for 48 hours, were subjected to the Annexin V/ PI staining and analyzed by flow cytometry. The quantification is shown in **E** (n=3 per group). (**F** and **G)** GBM22 and KNS42 cells were treated with CPI-613, ABT199, or combination of both for 48 hours, were subjected to the Annexin V/ PI staining and were analyzed by flow cytometry. The quantification is shown in **G** (n=3 per group). (**H)** GBM22 and KNS42 cells were transfected with non-targeting siRNA (siNT) or siRNA against OGDH, treated with ABT263 for 72h and analyzed for cell viability (n=4 per group). (**I)** OGDH and Actin western blots of GBM22 and KNS42 cells transfected with non-targeting siRNA (siNT) or with an siRNA against OGDH. (**J** and **K)** GBM22 and GBM12 cells were transduced with non-specific shRNA or shRNAs targeting OGDH, were treated with ABT263, and were analyzed by flow cytometry following staining with Annexin V/PI. The quantification is shown in **K**. Statistical significance was assessed ANOVA with Dunnett multiple comparison test in A, C, E, G, and K and by two-tailed Student t test in H. Data are shown as mean ± SD. *$p < 0.05$, **** $p < 0.001$.

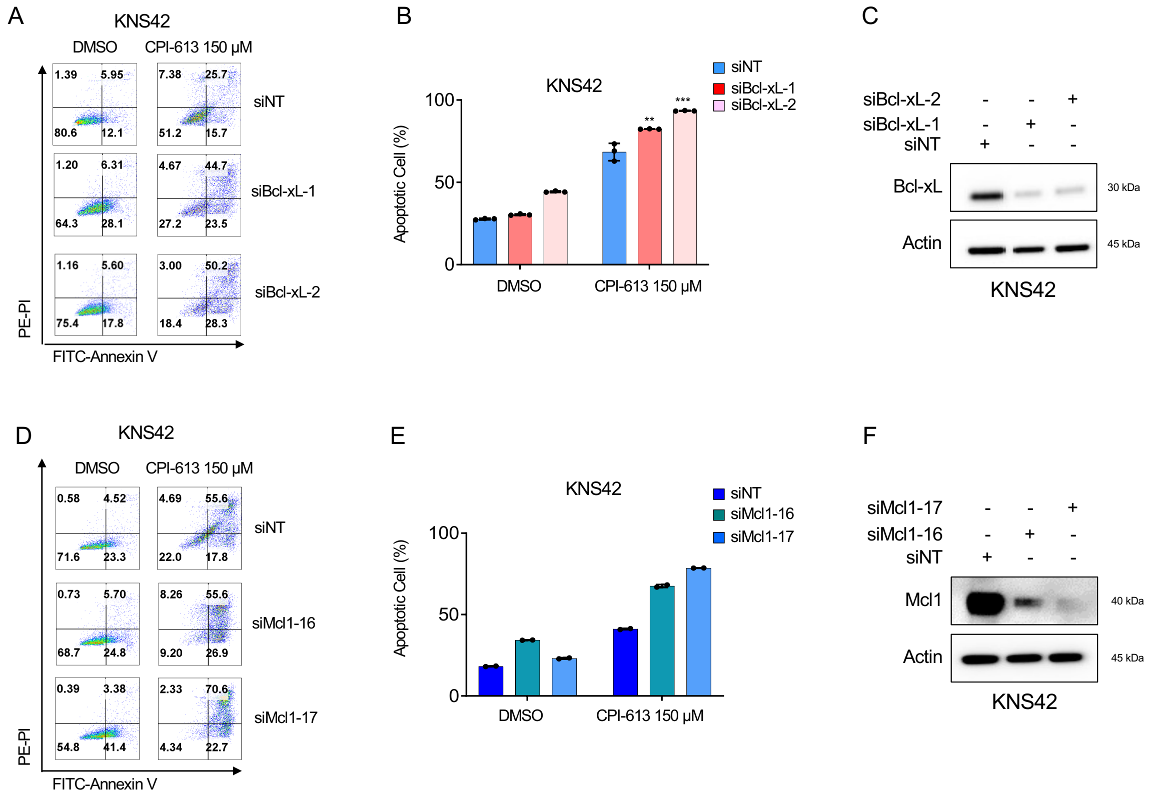

A
KNS42
DMSO
CPI-613 150 μM
1.39 5.95 80.6 12.1
7.38 25.7 51.2 15.7
siNT
1.20 6.31 64.3 28.1
4.67 44.7 27.2 23.5
siBcl-xL-1
1.16 5.60 75.4 17.8
3.00 50.2 18.4 28.3
siBcl-xL-2
PE-PI
FITC-Annexin V
B
KNS42
siNT
siBcl-xL-1
siBcl-xL-2
Apoptotic Cell (%)
100
50
0
**
***
DMSO
CPI-613 150 μM
C
siBcl-xL-2 - - +
siBcl-xL-1 - + -
siNT + - -
Bcl-xL
30 kDa
Actin
45 kDa
KNS42
D
KNS42
DMSO
CPI-613 150 μM
0.58 4.52 71.6 23.3
4.69 55.6 22.0 17.8
siNT
0.73 5.70 68.7 24.8
8.26 55.6 9.20 26.9
siMcl1-16
0.39 3.38 54.8 41.4
2.33 70.6 4.34 22.7
siMcl1-17
PE-PI
FITC-Annexin V
E
KNS42
siNT
siMcl1-16
siMcl1-17
Apoptotic Cell (%)
100
50
0
DMSO
CPI-613 150 μM
F
siMcl1-17 - - +
siMcl1-16 - + -
siNT + - -
Mcl1
40 kDa
Actin
45 kDa
KNS42

Supplementary Figure 7. Loss of Bcl-xL along with OGDH inhibition causes synthetic lethality in GBM cells. (A and **B)** KNS42 cells were transfected with non-targeting siRNA (siNT) or with siRNAs against Bcl-xL, were treated with CPI-613 and were analyzed by flow cytometry following Annexin V/PI staining. The quantification is shown in **B** (n=3 per group). (**C)** Bcl-xL and Actin western blots of KNS42 cells transfected with non-targeting siRNA (siNT) or with siRNA against Bcl-xL. (**D** and **E)** KNS42 cells were transfected with non-targeting siRNA (siNT) or with siRNAs against Mcl1, were treated with CPI-613 and were analyzed by flow cytometry following Annexin V/PI staining. The quantification is shown in **E** (n=2 per group). (**F)** Mcl1 and Actin western blots of KNS42 cells transfected with non-targeting siRNA (siNT) or with siRNAs against Mcl1. Statistical significance was assessed ANOVA with Dunnett multiple comparison test in B. Data are shown as mean ± SD. $^{**}p < 0.01$, $^{***} p < 0.001$.

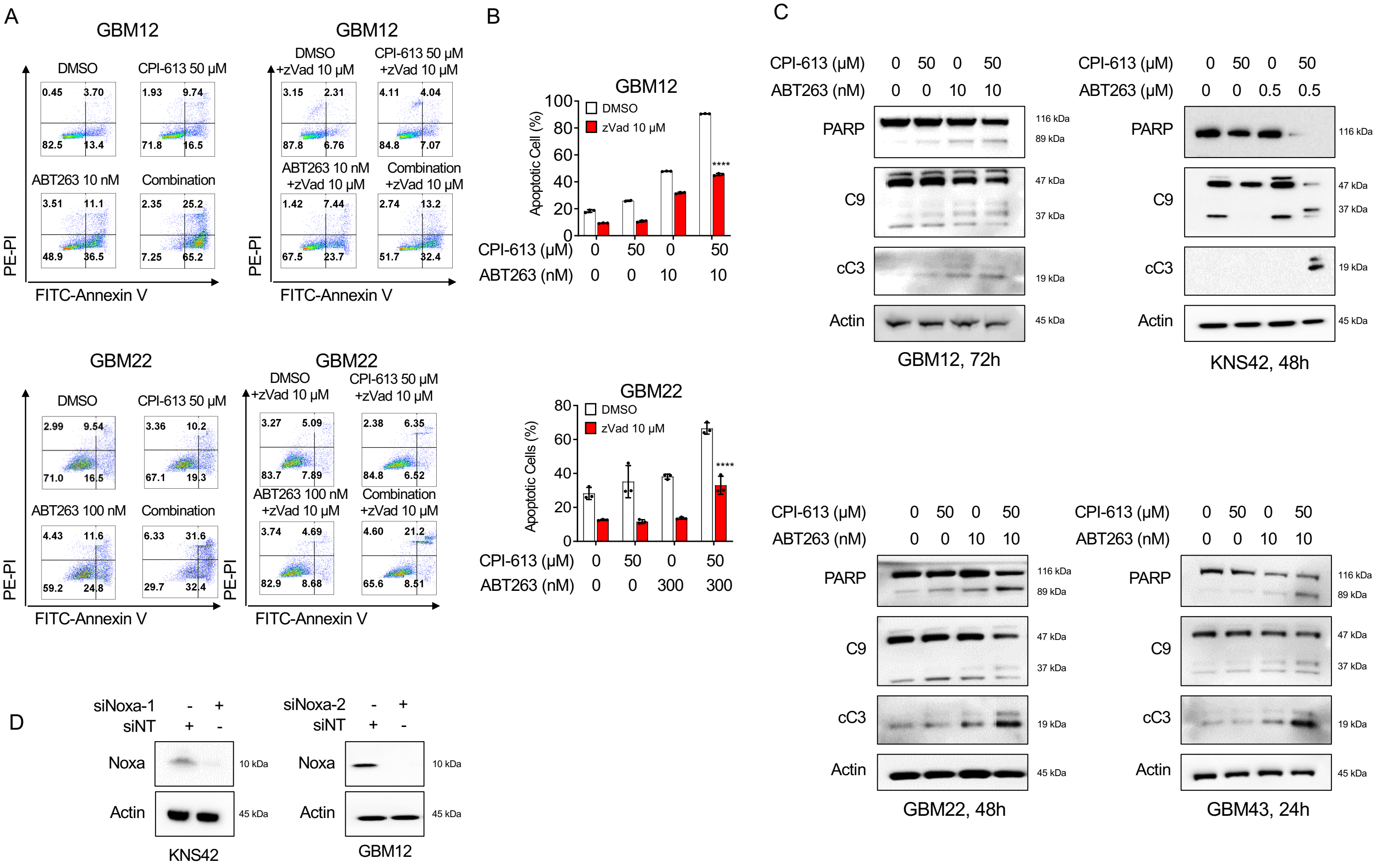

A
GBM12
DMSO
CPI-613 50 μM
ABT263 10 nM
Combination
DMSO +zVad 10 μM
CPI-613 50 μM +zVad 10 μM
ABT263 10 nM +zVad 10 μM
Combination +zVad 10 μM
PE-PI
FITC-Annexin V
GBM22
ABT263 100 nM
ABT263 100 nM +zVad 10 μM
B
Apoptotic Cell (%)
Apoptotic Cells (%)
DMSO
zVad 10 μM
CPI-613 (μM)
ABT263 (nM)
C
ABT263 (μM)
PARP
C9
cC3
Actin
116 kDa
89 kDa
47 kDa
37 kDa
19 kDa
45 kDa
GBM12, 72h
KNS42, 48h
GBM22, 48h
GBM43, 24h
D
siNoxa-1
siNoxa-2
siNT
Noxa
10 kDa
KNS42
GBM12

Supplementary Figure 8. The combination treatment of ABT263 and CPI-613 mediates enhanced caspase cleavage. (A and **B)** GBM12 and GBM22 cells were treated with CPI-613, ABT263, or combination of both in the presence or absence of 10 μM zVad for 48 hours, were subjected to flow cytometry following labeling with Annexin V/ PI. Quantification is shown in **B** (n=3 per group). (**C)** PARP, Caspase-9 (C9), cleaved caspase 3 (cC3) and actin western blots of GBM12, KNS42, GBM22, and GBM43 cells treated with CPI-613, ABT263, or the combination of both. Actin is used as a loading control. (**D)** Noxa and Actin western blots of KNS42 and GBM12 cells transfected with non-targeting siRNA or with an siRNA against Noxa. Statistical significance was assessed by two-tailed Student t test in B. Data are shown as mean ± SD. **** $p < 0.001$.

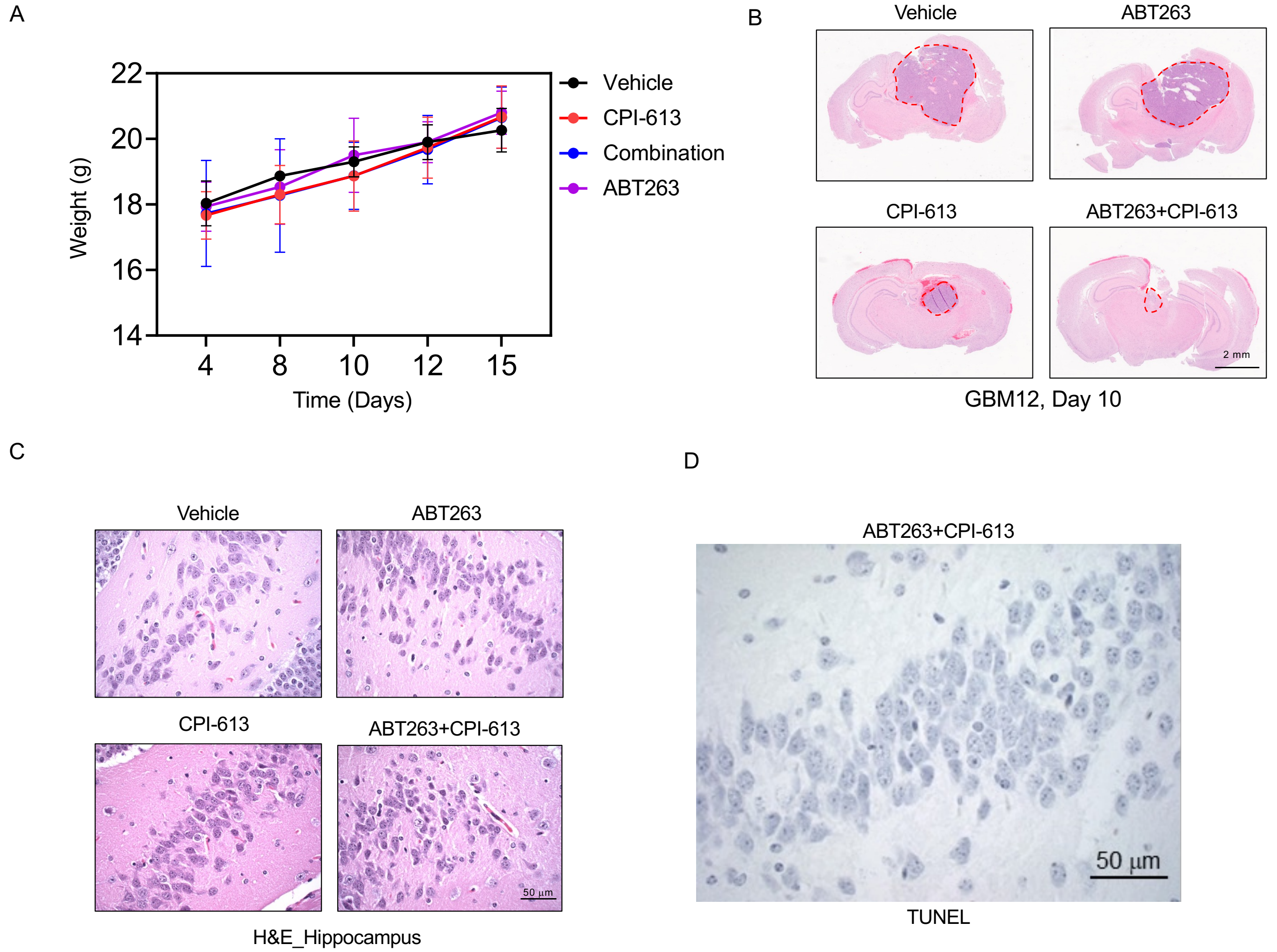
A
22
20
18
16
14
Weight (g)
4
8
10
12
15
Time (Days)
Vehicle
CPI-613
Combination
ABT263
B
Vehicle
ABT263
CPI-613
ABT263+CPI-613
2 mm
GBM12, Day 10
C
Vehicle
ABT263
CPI-613
ABT263+CPI-613
50 μm
H&E_Hippocampus
D
ABT263+CPI-613
50 μm
TUNEL

Supplementary Figure 9. The combination treatment of ABT263 and CPI-613 reveals anti-glioma activity in vivo. (A) Nude mice were randomly assigned to the following groups: vehicle, CPI-613, ABT263, and the combination of both. **(B)** GBM12 cells were implanted in the right striatum of nude mice. After establishment of tumors, four groups were randomly assigned: vehicle, CPI-613, ABT263, and the combination of both. Seven days after the implantation, mice were treated three times per week. The brain tumors were fixed and stained with H&E. Scale bar: 2 mm. **(C)** GBM12 cells were implanted in the right striatum of nude mice. After establishment of tumors, four groups were randomly assigned: vehicle, CPI-613, ABT263, and the combination of both. Seven days after the implantation, mice were treated three times per week. The brains were fixed and stained with H&E. Shown are hippocampal neurons from the various treatment groups. **(D)** GBM12 cells were implanted in the right striatum of nude mice. After establishment of tumors, four groups were randomly assigned: vehicle, CPI-613, ABT263, and the combination of both. Seven days after the implantation, mice were treated three times per week. The brains were fixed and stained with TUNEL. Shown are hippocampal neurons from the combination treatment group (ABT263+CPI-613). Scale bar: 50 μm. Data are shown as mean ± SD.

Supplementary Table 1

Primer sequences for real time PCR and chromatin immunoprecipitation qPCR

qPCR primer ATF3 F	CGCTGGAATCAGTCACTGTCAG
qPCR primer ATF3 R	CTTGTTTCGGCACTTTGCAGCTG
qPCR primer ATF4 F	TTCTCCAGCGACAAGGCTAAGG
qPCR primer ATF4 R	CTCCAACATCCAATCTGTCCCG
qPCR primer Noxa F	CTGGAAGTCGAGTGTGCTACTC
qPCR primer Noxa R	TGAAGGAGTCCCCTCATGCAAG
qPCR primer Bcl2_F	ATCGCCCTGTGGATGACTGAGT
qPCR primer Bcl2 R	GCCAGGAGAAATCAAACAGAGGC
qPCR primer Bcl-xL F	GCCACTTACCTGAATGACCACC
qPCR primer Bcl-xL R	AACCAGCGGTTGAAGCGTTCCT
qPCR primer Mcl1 F	CCAAGAAAGCTGCATCGAACCAT
qPCR primer Mcl1 R	CAGCACATTCCTGATGCCACCT
ChIP qPCR primer Noxa F	GCTGTTGCATCAGACGATTATAC
ChIP qPCR primer: Noxa R	CAACTTAGGCATGGTCACATTT
