# Supplementary material for: OGDH and Bcl-xL loss causes synthetic lethality in glioblastoma
Source: JCI Insight. 2024 Mar 14;9(8):e172565. doi: 10.1172/jci.insight.172565 (PMC11141877; doi:10.1172/jci.insight.172565)

Figure 1D

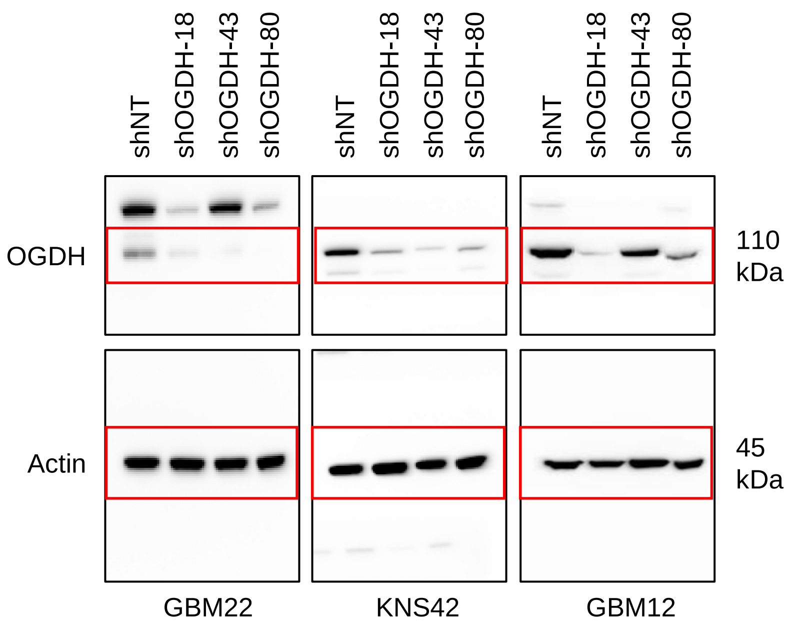
shNT
shOGDH-18
shOGDH-43
shOGDH-80
shNT
shOGDH-18
shOGDH-43
shOGDH-80
shNT
shOGDH-18
shOGDH-43
shOGDH-80
OGDH
110 kDa
Actin
45 kDa
GBM22
KNS42
GBM12

Figure 2A

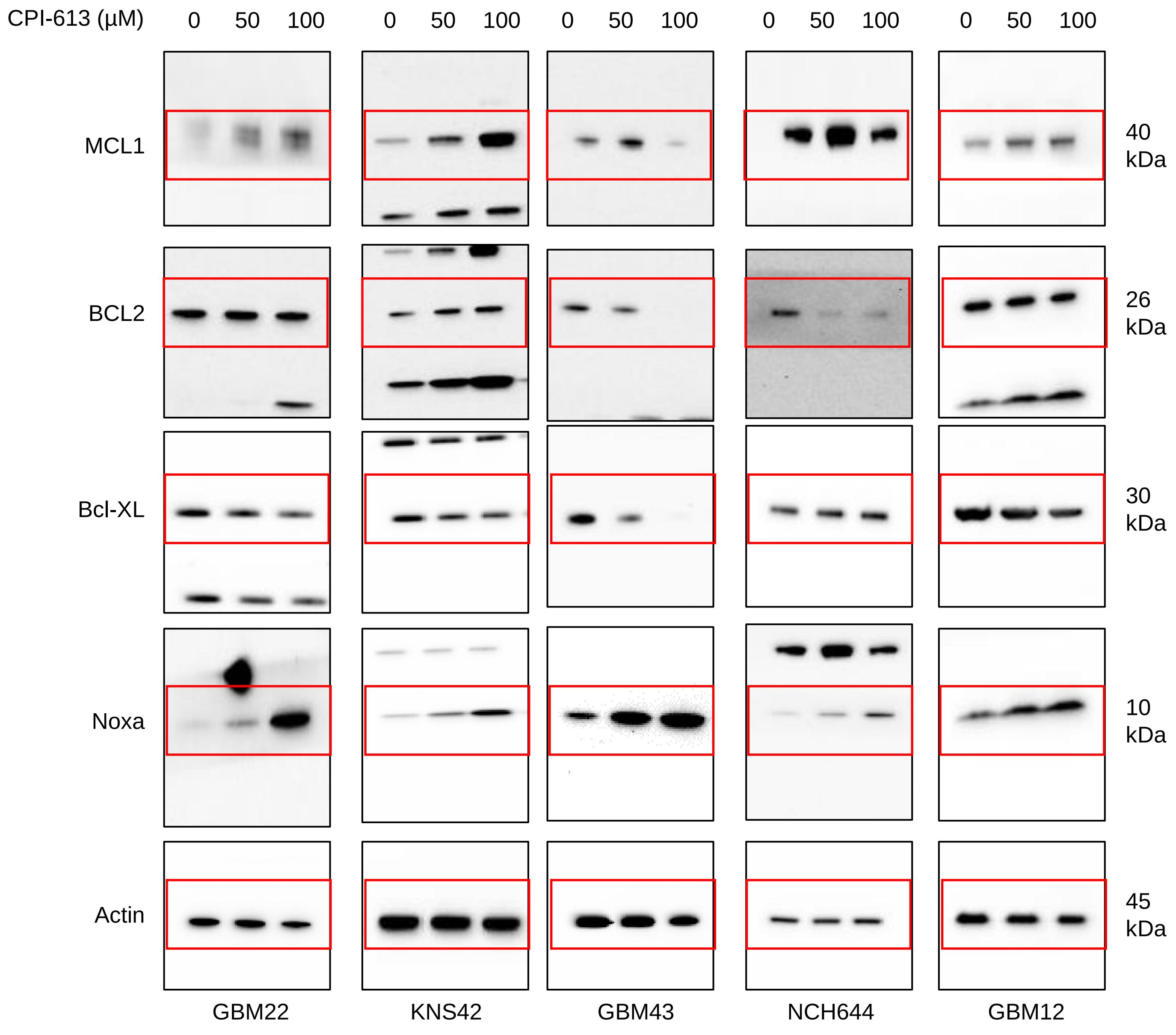

CPI-613 (µM)
0
50
100
0
50
100
0
50
100
0
50
100
0
50
100
MCL1
BCL2
Bcl-XL
Noxa
Actin
40 kDa
26 kDa
30 kDa
10 kDa
45 kDa
GBM22
KNS42
GBM43
NCH644
GBM12

Figure 4B

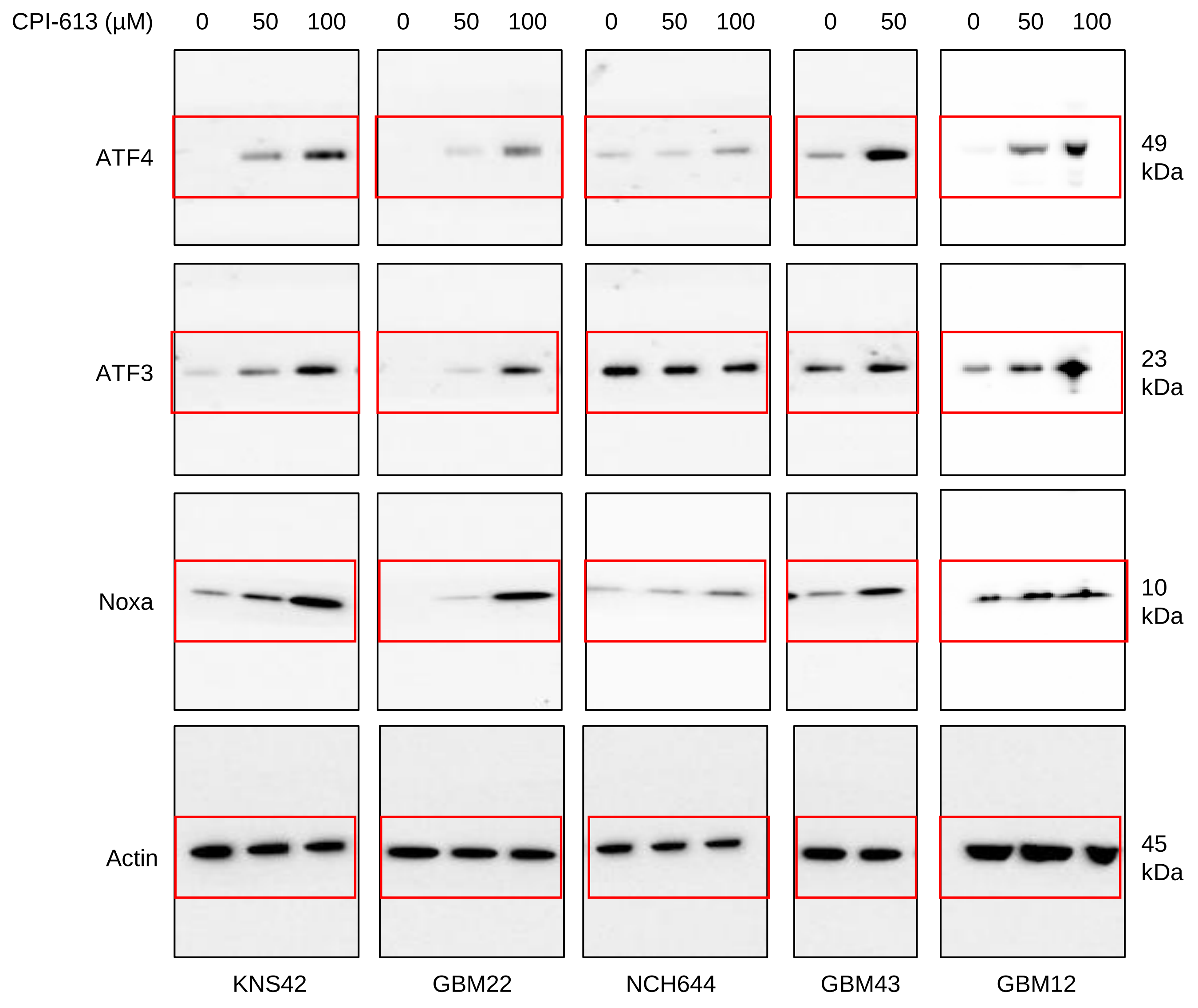

CPI-613 (µM)
0
50
100
0
50
100
0
50
100
0
50
0
50
100
ATF4
ATF3
Noxa
Actin
49 kDa
23 kDa
10 kDa
45 kDa
KNS42
GBM22
NCH644
GBM43
GBM12

Figure 4D

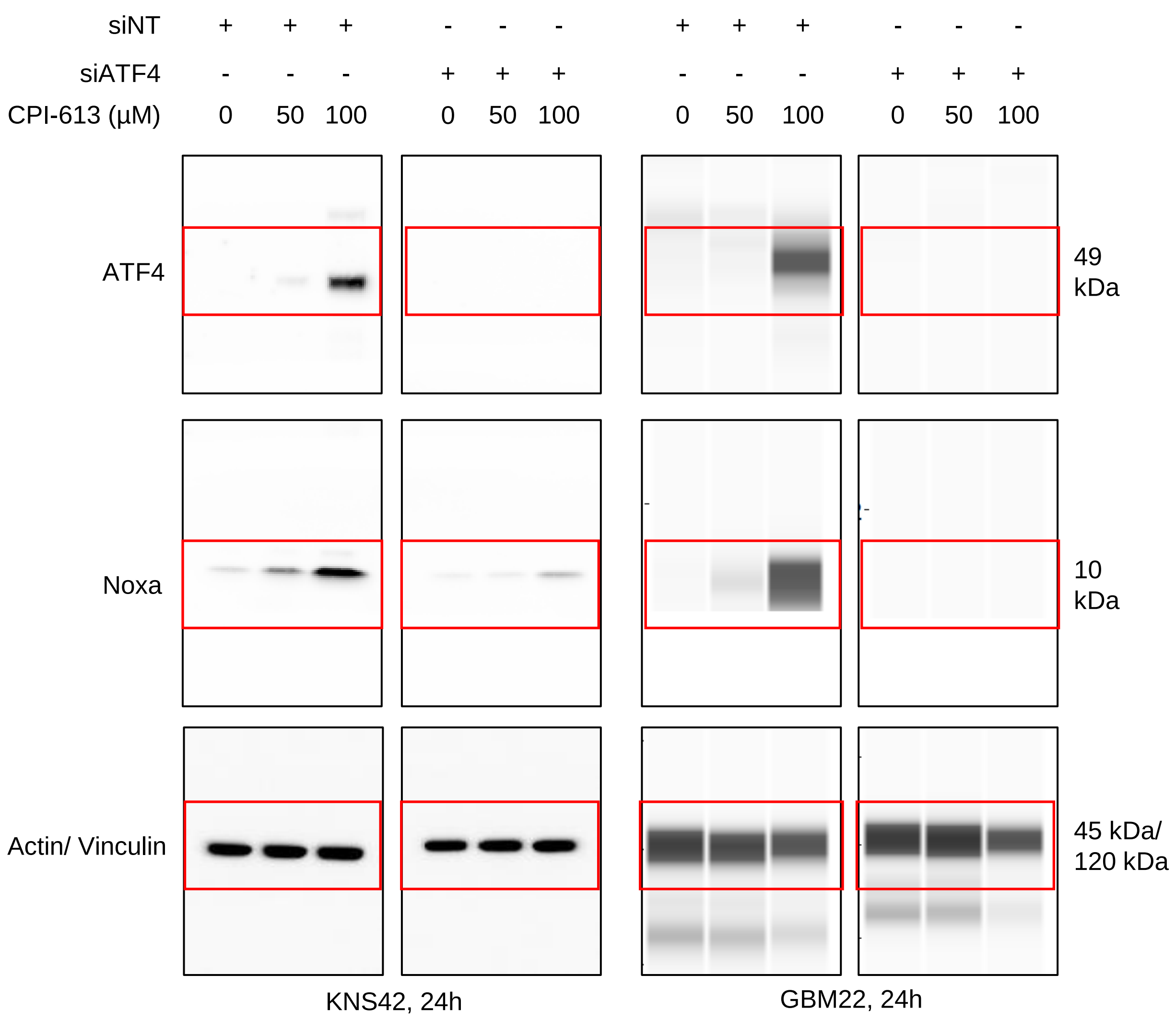

siNT + + + - - - + + + - - -
siATF4 - - - + + + - - - + + +
CPI-613 (µM) 0 50 100 0 50 100 0 50 100 0 50 100
ATF4
49 kDa
Noxa
10 kDa
Actin/ Vinculin
45 kDa/ 120 kDa
KNS42, 24h
GBM22, 24h

Figure Supplement 2B

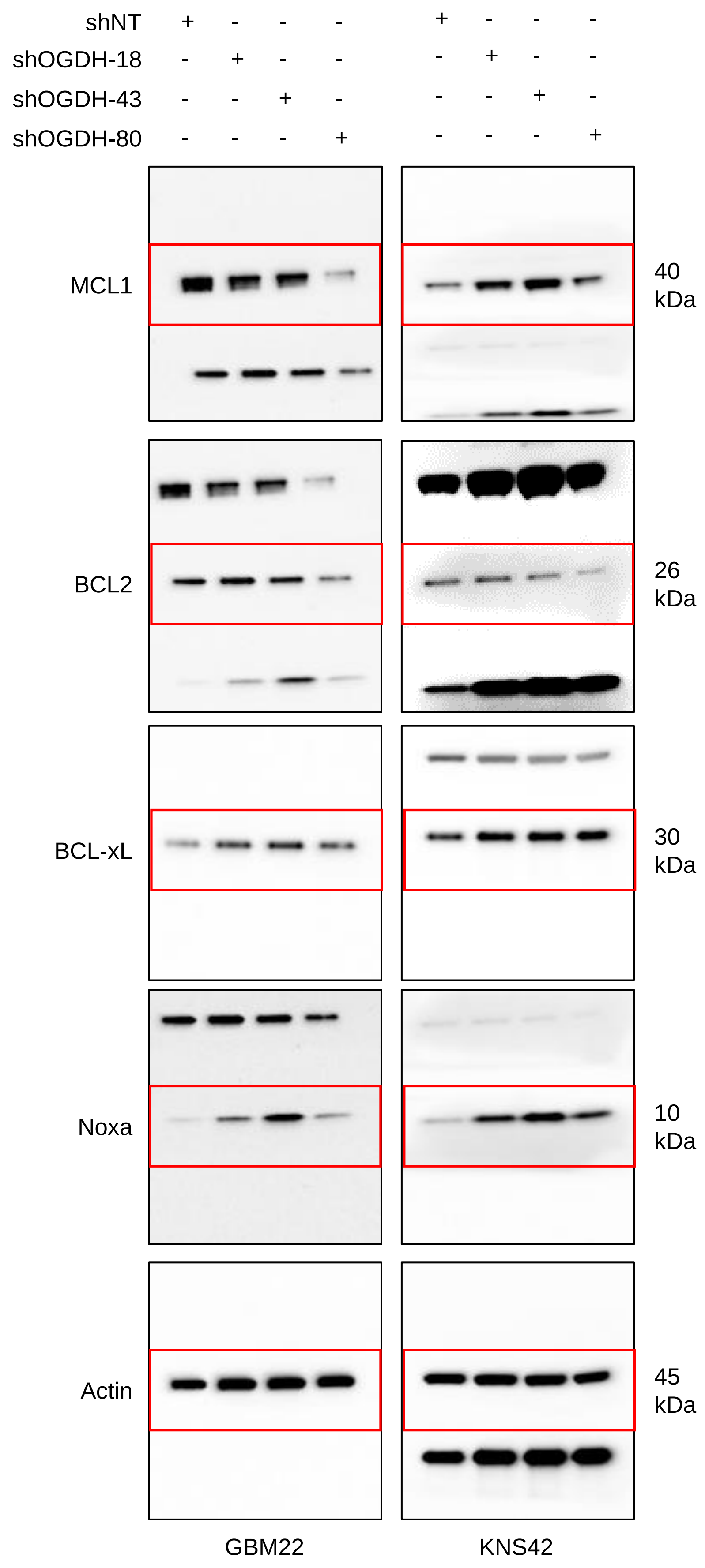

Figure Supplement 2C

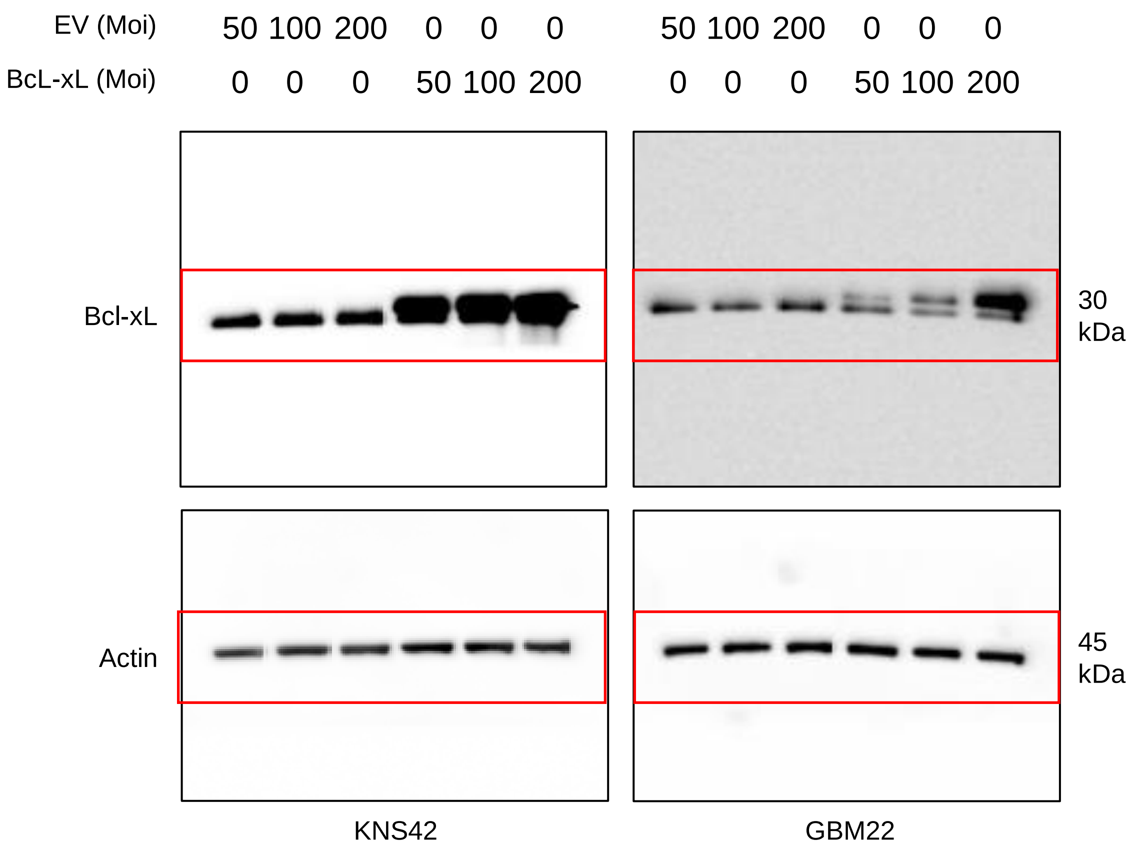

Figure Supplement 2E

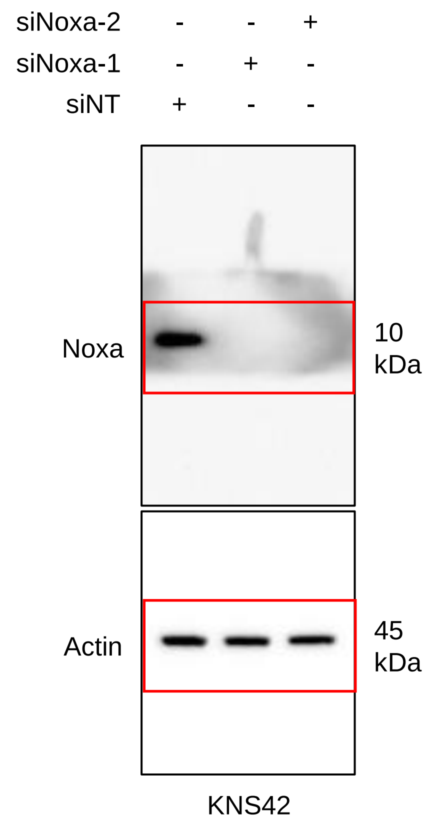

Figure Supplement 2H

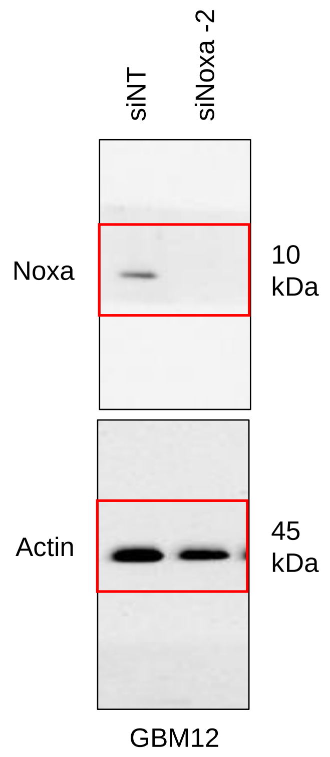

Figure Supplement 5A

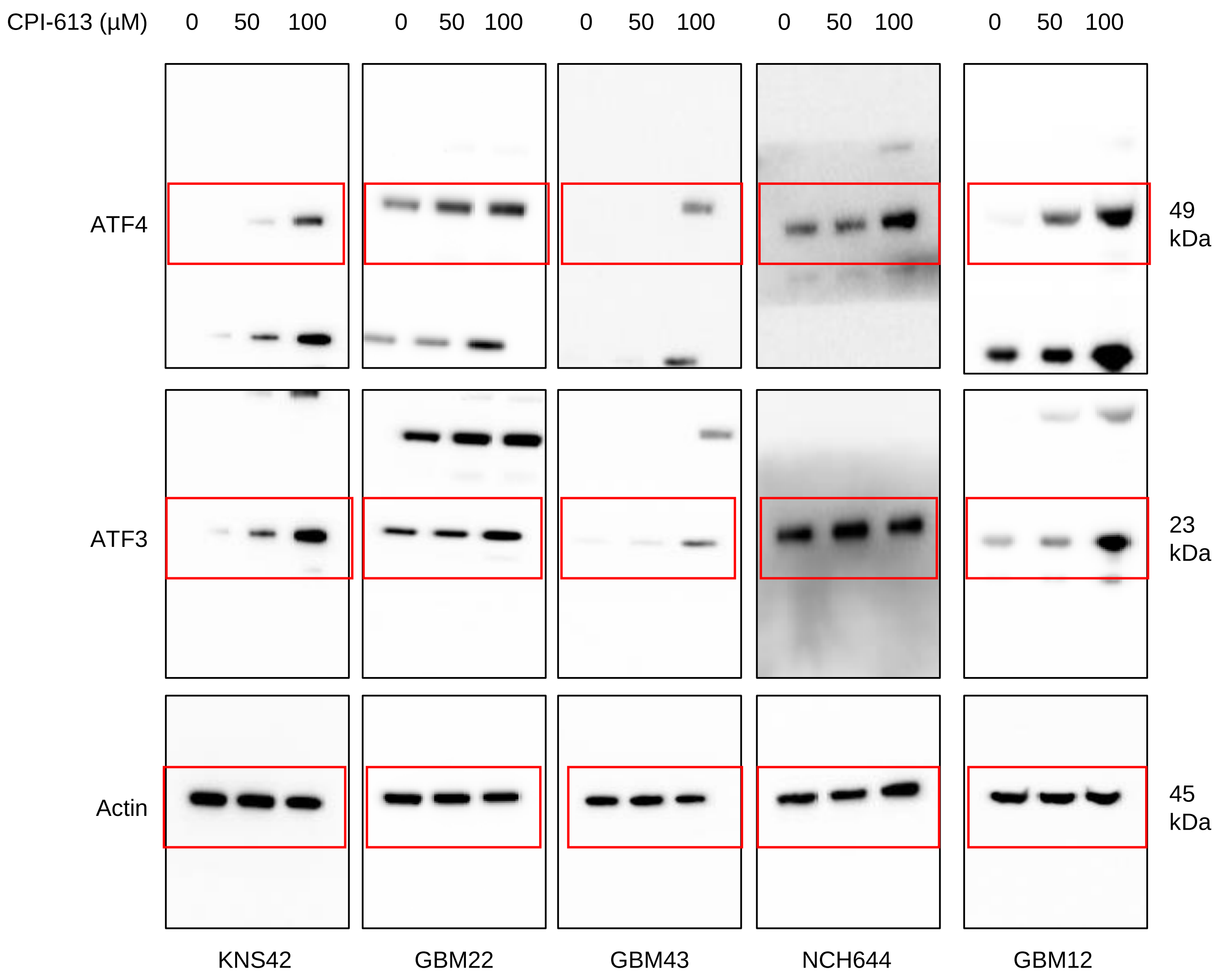

Figure Supplement 5B

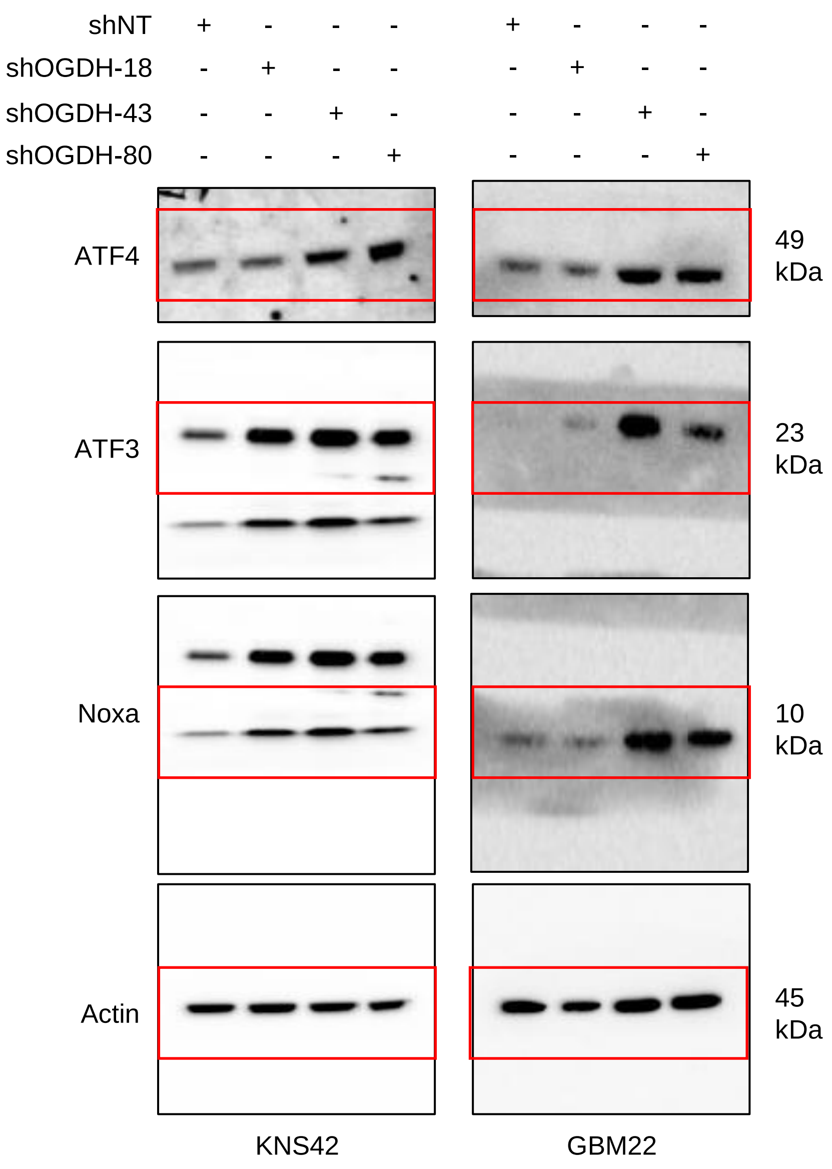

Figure Supplement 5C

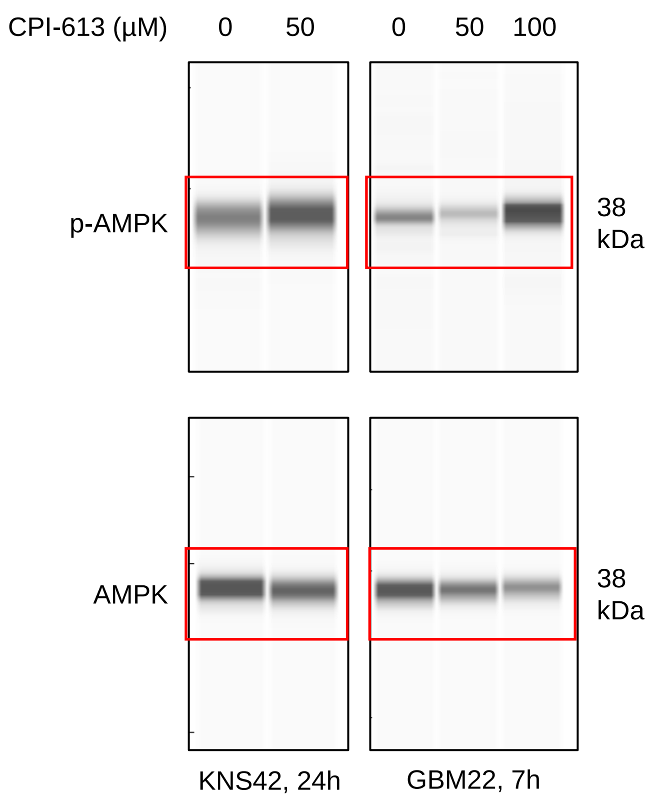

Figure Supplement 5D

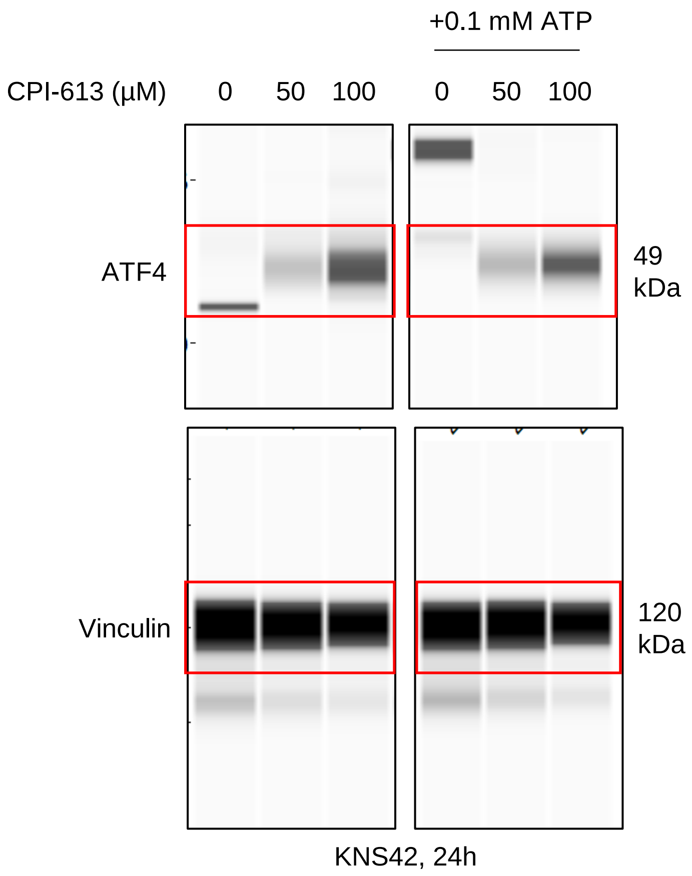

Figure Supplement 5E

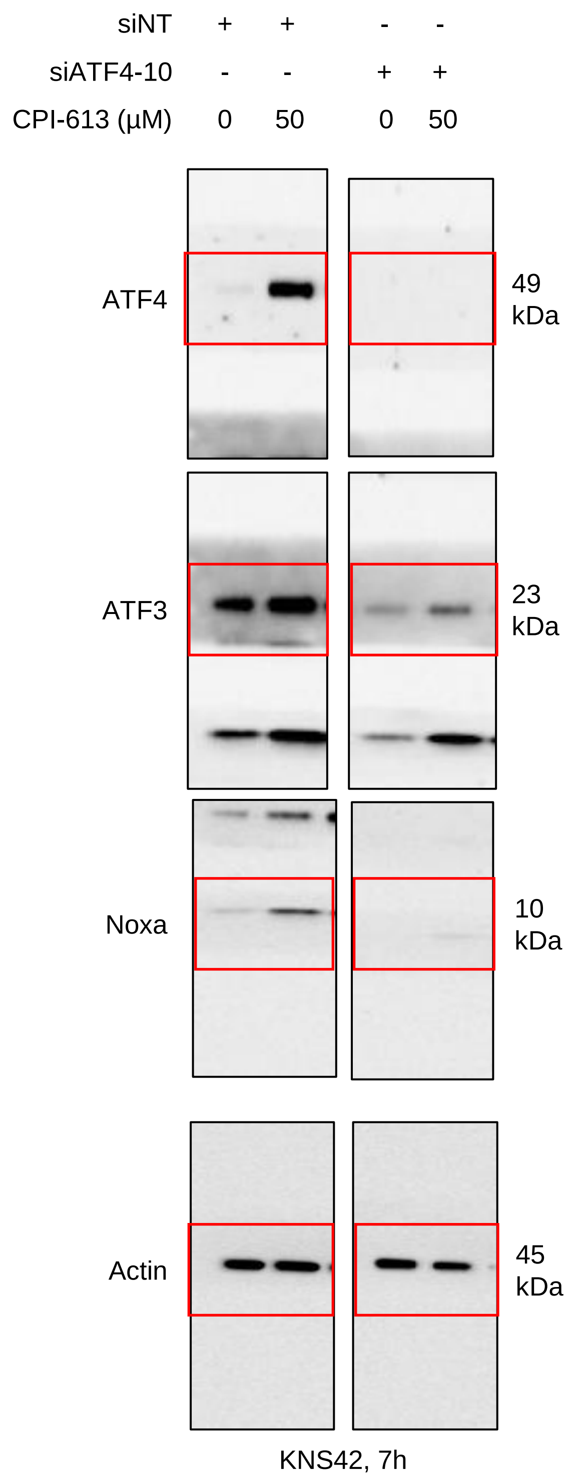


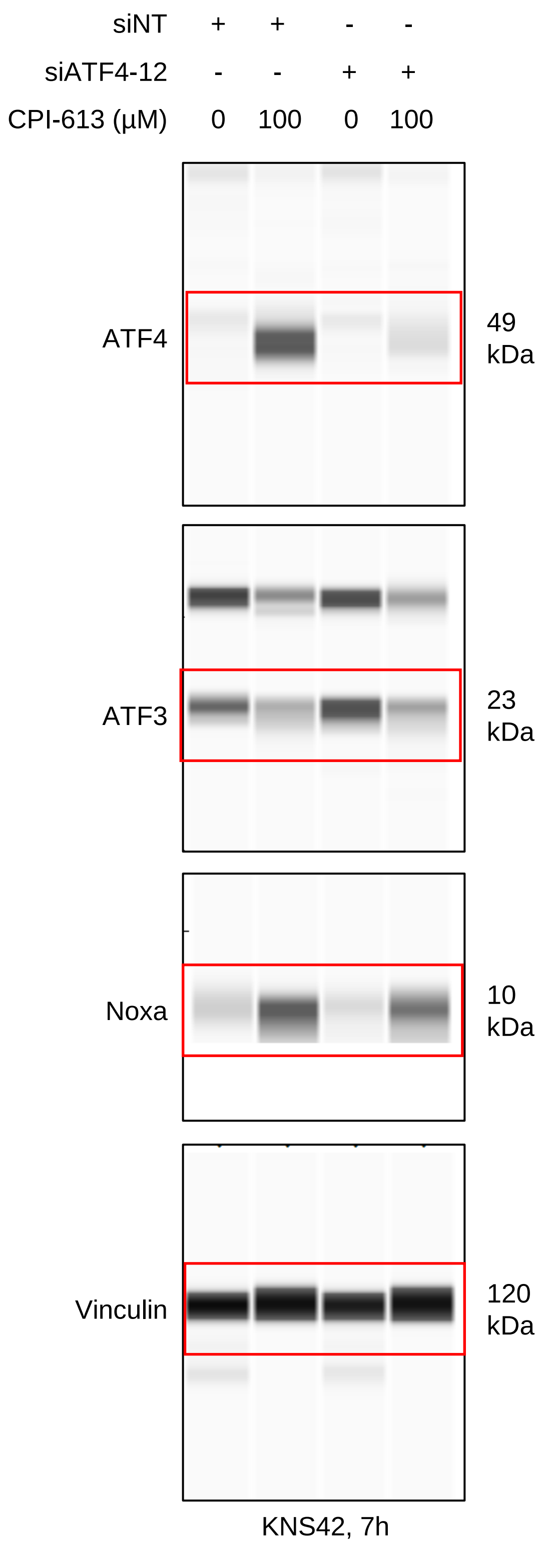

# Figure Supplement 6I

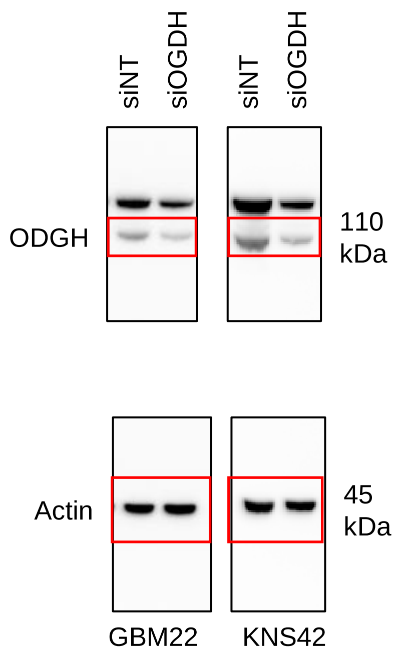

Figure Supplement 7C

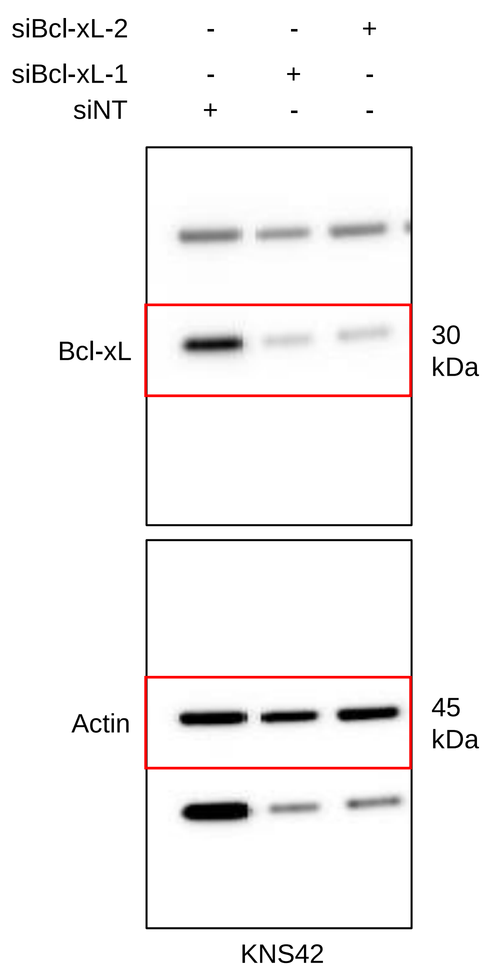

Figure Supplement 7F

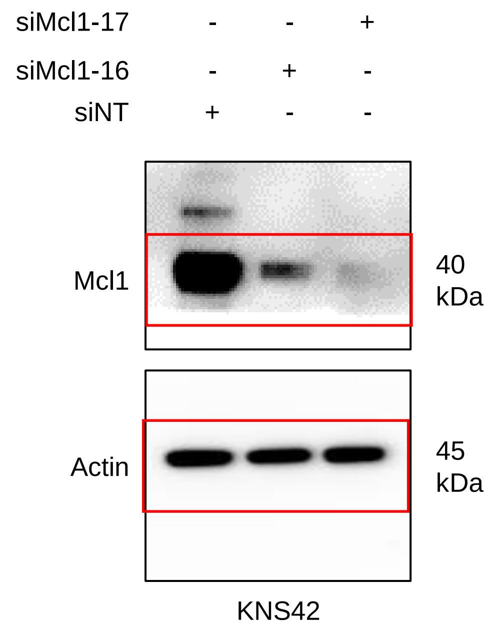

Figure Supplement 8C

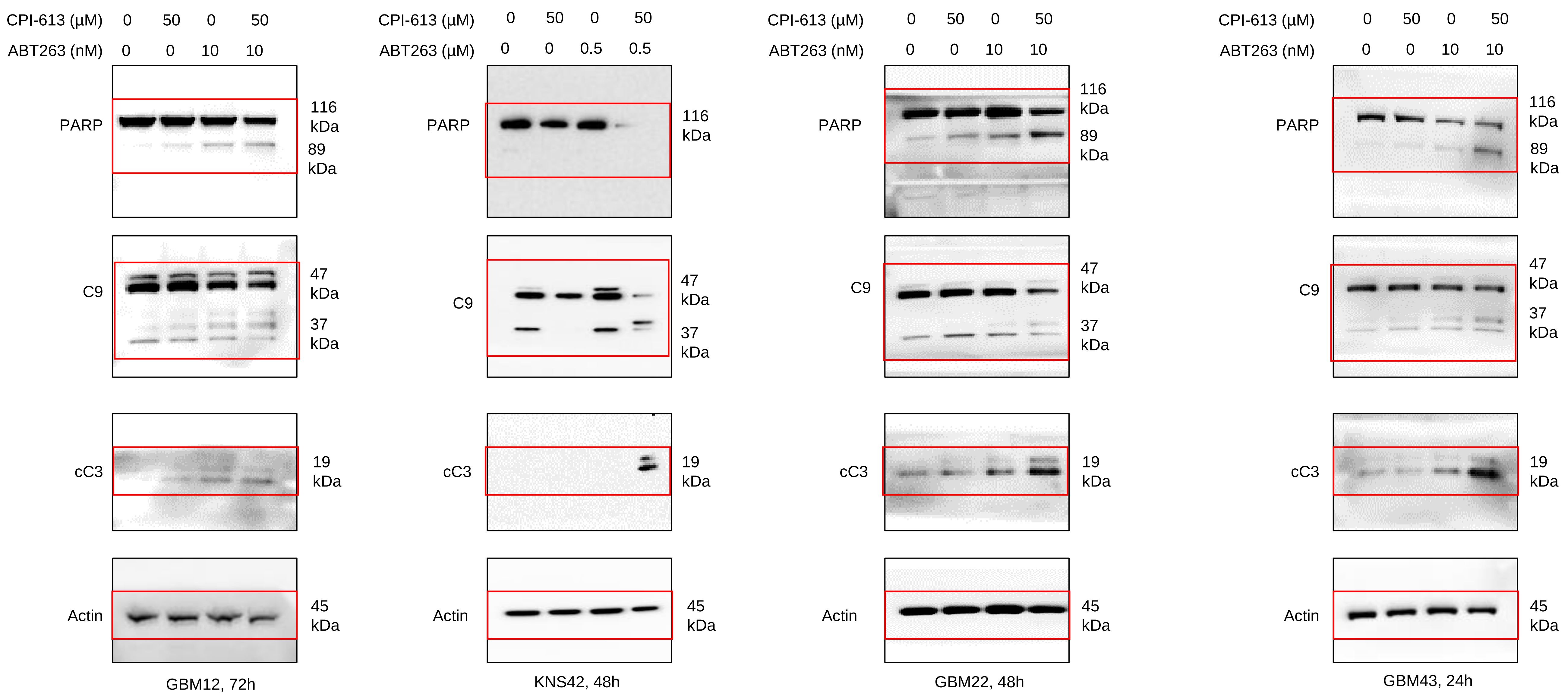

Figure Supplement 8D

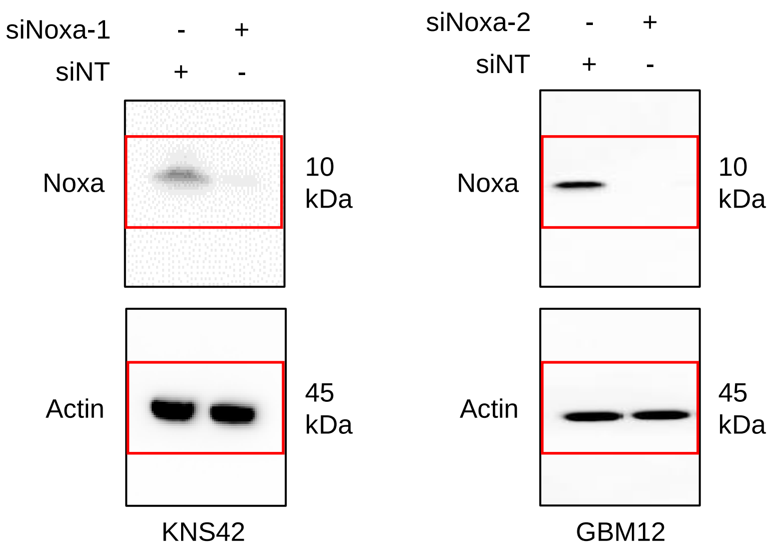

Supplement: Unedited blot and gel images [file jciinsight-9-172565-s119.pdf]
